# Detection of Sugar Syrups in Honey Using Untargeted Liquid Chromatography–Mass Spectrometry and Chemometrics

**DOI:** 10.3390/metabo14110633

**Published:** 2024-11-16

**Authors:** Jule Hansen, Christof Kunert, Kurt-Peter Raezke, Stephan Seifert

**Affiliations:** 1 Hamburg School of Food Science, Institute of Food Chemistry, University of Hamburg, Grindelallee 117, 20146 Hamburg, Germany; 2 Eurofins Food Integrity Control Services GmbH, Berliner Str. 2, 27721 Ritterhude, Germany

**Keywords:** syrup adulteration, honey, routine analysis, untargeted LC-MS, machine learning, random forest, classification, regression

## Abstract

**Background**: Honey is one of the most adulterated foods worldwide, and several analytical methods have been developed over the last decade to detect syrup additions to honey. These include approaches based on stable isotopes and the specific detection of individual marker compounds or foreign enzymes. Proton nuclear magnetic resonance (^1^H-NMR) spectroscopy is applied as a rapid and comprehensive screening method, which also enables the detection of quality parameters and the analysis of the geographical and botanical origin. However, especially for the detection of foreign sugars, ^1^H-NMR has insufficient sensitivity. **Methods**: Since untargeted liquid chromatography–mass spectrometry (LC-MS) is more sensitive, we used this approach for the detection of positive and negative ions in combination with a recently developed data processing workflow for routine laboratories based on bucketing and random forest for the detection of rice, beet and high-fructose corn syrup in honey. **Results**: We show that the distinction between pure and adulterated honey is possible for all three syrups, with classification accuracies ranging from 98 to 100%, while the accuracy of the syrup content estimation depends on the respective syrup. For rice and beet syrup, the deviations from the true proportion were in the single-digit percentage range, while for high-fructose corn syrup they were much higher, in some cases exceeding 20%. **Conclusions**: The approach presented here is very promising for the robust and sensitive detection of syrup in honey applied in routine laboratories.

## 1. Introduction

Honey is one of the most adulterated foods in the world, adulterated through false labelling of the geographical or botanical origin, illegal processing and the addition of sugar syrup [1,2]. The latter is economically motivated by the high price of honey compared to syrups made from cheaper sources such as rice or beets, as producers can increase their profits significantly even by adding only small amounts of syrup. However, in the European Union (EU), it is prohibited to add any food ingredient, including food additives or other additions to honey (Honey Directive, Council Directive 2001/110/EC relating to honey, Annex II), and the EU coordinated action From the Hives showed that a significant proportion of the honey imported into the EU is suspicious or has been found not to comply with the provisions of the Honey Directive [3]. Sensitive and reliable analytical methods are therefore required to detect syrup adulteration in honey to ensure compliance with regulations and the trustworthiness of beekeepers.

Currently, syrup in honey is detected by commercial and official laboratories using ^13^C/^12^C stable carbon isotope ratio mass spectrometer coupled with elemental analyzer liquid chromatography (^13^C-EA/LC-IRMS) [4], untargeted proton nuclear magnetic resonance (^1^H-NMR) [5] and targeted Liquid Chromatography–Mass Spectrometry (LC-MS). In the latter, known marker molecules defined for specific syrups are detected, which is time-consuming because extensive sample preparation and multiple analyses are required [6]. Furthermore, it is not trivial to find suitable marker molecules due to the similarity of honeys and sugar syrups and the high variance within the honey samples [7]. This diversity is due to the fact that the diet of bees is less well defined than that of other animals or plants, for example because they collect pollen and nectar within a 5 km radius of their hive [8,9]. Furthermore, the suitable markers found, which are stored in marker databases, must constantly be updated, as fraudsters are aware of the substances being tested and adapt their fraud methods accordingly [5,10]. This has also been demonstrated in a comprehensive study of economically motivated adulteration based on a large number of specific cases [11].

^1^H-NMR is often applied untargeted, which means that the entire ^1^H-NMR data of the samples are analysed with multivariate approaches. These can be supervised or unsupervised, i.e., the membership of the samples to the targeted grouping can be included into the analysis or not. Principal Component Analysis (PCA) is an unsupervised approach that generates latent principal components used to analyse the main variances in the data [12,13,14]. Supervised approaches such as Random Forest (RF) and Support Vector Machine train models based on specific differences between predefined classes [15,16,17,18], e.g., regarding pure or adulterated honey [5,19,20]. RF is a non-parametric ensemble learning algorithm based on multiple binary decision trees, which has several advantages for this application. Because each of the decision trees is trained on a different subset of the samples, called bootstrap sample, the remaining samples, called out-of-bag samples, can be used to generate an independent error, called out-of-bag (OOB) error [21]. This means that no additional data is necessary to validate the performance of the obtained model. The other advantages are its easy adaptation to imbalanced training data and the minimal need for parameter tuning [22], which is why ^1^H-NMR in combination with RF has been applied for the authentication of various foods [18,23,24,25,26,27].

Even though the use of ^1^H-NMR as an untargeted method offers the advantages of relatively simple sample preparation, the possibility of quantifying metabolites and a high degree of experimental reproducibility, this approach has the disadvantage of a rather low sensitivity [28]. This means that syrup additions are often only detected in comparatively large quantities [5]. Therefore, it would be a great asset for honey monitoring laboratories to use the more sensitive LC-MS method as an untargeted approach.

Untargeted LC-MS is already used successfully for the authentication of various foods [29,30,31,32,33], including honey [7,34,35,36]. In these applications, however, usually only samples measured within a short period of time using one single instrument [37] can be analysed together due to instrumental shifts and the resulting need to define peaks during processing [38]. The lack of robustness is primarily due to the varying age and degree of contamination of the devices and the columns used [39]. This causes a slightly different interaction between the compounds and the columns and, hence, results in measurement-based differences in the spectra if they are not obtained at the same time with the same device [40]. These circumstances are the reason why current LC-MS data processing workflows such as vendor-specific software (e.g., Compound Discoverer^TM^ Software [41]) or the framework for processing and visualizing LC-MS data xcms [42,43,44] include a so-called retention time alignment step. Additionally, in common data processing strategies, a correspondence step is included to match the detected and aligned chromatographic peaks between the different spectra to generate a common peak list, which is unique for each analysis. Therefore, it is currently not possible to analyse untargeted LC-MS spectra that were processed separately. For long-term application, this means an immense computational effort, as the entire data set would have to be reprocessed each time a newly analysed sample is to be classified or new authentic data are added. In routine analysis, several hundred samples are analysed every week and it is therefore currently very difficult to apply untargeted LC-MS in this field. This is why untargeted LC-MS is often used to identify marker molecules for specific groups, which are then applied in targeted analyses [6,38,45,46]. However, to use the full potential of the large amounts of data generated, the long-term use and separate processing of the LC-MS data are necessary. We have recently developed a data processing approach, Bucketing of Untargeted LC-MS Spectra (BOULS), which makes this possible, as demonstrated by the authentication of the geographical origin of honey [47].

In this paper we evaluate LC-MS-based approaches in combination with BOULS and multivariate analysis for the detection of different syrups used in a routine laboratory for the sensitive analysis of honey. For this, we analysed 34 North German honey samples mixed with rice, beet and high-fructose corn syrup with different LC approaches. For the detection of polar and non-polar compounds, Hydrophilic Interaction Liquid Chromatography (HILIC) in negative ionization mode and Reversed Phase (RP) chromatography in positive ionization mode are used, respectively [8,35], and we evaluate whether one of these approaches should be used individually, together or in addition with fragmentation data.

## 2. Materials and Methods

### 2.1. Sample Preparation

For authentication, mainly to ensure that the North German honey samples were actually free of foreign sugars, they were analyzed using pollen microscopy (pollen identification and determination of the relative pollen content, DIN 10760 mod. (2002–05)), sensory (ICS SOP 520–02 (2018–08)), ^13^C-EA/LC-IRMS [4], untargeted proton nuclear magnetic resonance (^1^H-NMR) [5] and targeted LC-HRMS [35]. As foreign sugar was detected in one of the 35 samples, it was excluded from the following analysis. For the analysis of the remaining 34 honey samples, 1 g of honey sample, as well as 1 g of rice, beet and high-fructose corn syrup was dissolved in 9.8 mL of deionised water and centrifuged. Subsequently, to produce adulterated honey samples, each syrup was added to each honey, creating mixtures with 5, 10, 20, 50 and 80% *v*/*v* syrup content. The 5 mixtures of each honey and syrup, as well as samples of the pure honey and syrup were stored at −20 °C until the measurement.

### 2.2. LC-MS Analysis

The samples were analysed in a randomized order using three different LC-HRMS devices (Thermo Scientific™ UltiMate™ 3000 systems coupled to Thermo Scientific™ QExactive™ Hybrid Quadrupole-Orbitrap™ High-Resolution Mass Spectrometers, Thermo Scientific™, Bremen, Germany). Each sample was analysed using two different LC approaches. Due to preliminary tests, HILIC (Accucore-150-Amide-HILIC, 150 × 2.1 mm, Thermo Scientific™, Bremen, Germany) was applied in negative mode to analyse polar compounds, and non-polar compounds were analysed using RP (Hypersil Gold C18, 150 × 2.1 mm, Thermo Scientific™, Bremen, Germany) chromatography in positive mode. For both chromatographic methods, the mobile phases acetonitrile and water were used with acetic acid as modifier. The ionization was performed using electrospray ionization (ESI) and the data were acquired in profile mode. For the mass spectrometric analysis, the variable data-independent acquisition (vDIA) approach developed by Thermo Scientific^TM^ Orbitrap^TM^ was applied using MS/MS precursor isolation windows of differing mass ranges, covering the entire mass range of 100–1500 Da (MS1) of the preceding full scan [48]. Ions of the following masses were fragmented: 100–200, 200–300, 300–400, 400–500, 500–1000 and 1000–1500 Da and the fragments were detected in the ranges of 50–225, 50–330, 50–430, 50–535, 69–1045, and 104–1555 Da, respectively [35].

This experimental setup resulted in 4 different data sets: HILIC full-scan data, HILIC full-scan and fragment data, RP full-scan data, RP full-scan and fragment data.

### 2.3. Data Analysis

The data processing and analysis were carried out in R (version 4.4.1) [49] using the BOULS approach [47] (https://github.com/AGSeifert/BOULS, accessed on 11 November 2024), requires Linux OS), which is based on the xcms workflow and uses the same functions for data import and chromatographic peak detection [50]. For data import, the thermo-specific raw files of the HILIC and RP data of each analysed sample were converted to open-format mzML files using MSConvert, which is part of the ProteoWizard software package (version 3.0.21078-7da1f1136 (developer build)), using the filter peakPicking to convert the profile data into centroided data [51]. The Bioconductor package mzR (version 2.26.0) was used for data import into R [51,52,53,54]. The package MSnbase (version 2.18.0) [55,56] was used to load and store the data in an object that was compatible with the xcms package (version 3.14.0) [42,44]. The centWave algorithm [44] was used for peak detection with the parameters peakwidth of 15 s and ppm of 5 Da. Retention time alignment was performed using the obiwarp method (bin size of 0.1 and the localAlignment set to TRUE). The data of one honey were used for retention time alignment and the 50% dilution with the respective syrup was used as center spectrum [57]. Subsequently, the data were processed using the BOULS approach with bucket sizes of 20 s in the retention time dimension and 2 Da in the mass dimension. The data were normalized by dividing the summarized intensities of each bucket by the sum of intensities of all signals, as previously determined during optimization of data processing with BOULS [47].

For multivariate data analysis, the processed HILIC and RP data were analyzed separately and after a low-level data fusion. This resulted in 6 analyses using HILIC full-scan data (42,000 variables), HILIC full-scan and fragment data (87,060 variables), RP full-scan data (67,200 variables), RP full-scan and fragment data (85,344 variables), the fused data set of HILIC and RP full-scan data (109,200 variables) and the fused data set of HILIC and RP full-scan and fragment data (172,404 variables). In this manuscript, the results of the data fusion of HILIC and RP full-scan data for the three syrups are shown. Due to the similarities, the results of the remaining data are shown in the Supplementary Materials.

For PCA, the stats package [58,59,60] (version 4.4.1) was used and the scatter plots were visualized using the package ggplot2 [61] (version 3.5.1). To visualize the main variance in the data, the first and second principal components were used. For RF analysis, nine honey samples were randomly selected and the respective data were used as test data for all analyses. The data of the other 25 honey samples were used for training of all RF classification and regression models using the ranger function of the R package ranger [62] (version 0.16.0). One sample (50% syrup proportion) was removed from the high-fructose corn syrup training data set, as the RP spectrum did not contain any signals, presumably due to injection problems during the measurement. For classification and regression, the number of trees (num.trees) was set to 5000 and default settings for mtry (205 for HILIC, 259 for RP and 330 for the fused data set) and min.node.size (1 for classification and 5 for regression) were chosen. As the data sets contained less data from pure honey samples than from adulterated samples, for classification, the case.weights parameter was chosen according to the size of the respective classes to compensate for class imbalance. The Root Mean Square Error (RMSE) was used to evaluate the precision of the predictions of the RF regression models (Metrics package, version 0.1.4 [63]).

## 3. Results and Discussion

In this study, untargeted LC-MS data in combination with multivariate data analysis were applied for analysis as in a routine laboratory to detect three different sugar syrups in honey. Various approaches for mass spectrometric analysis with several devices were used for this purpose and compared to verify the detection of syrups over a wide range of proportions and at comparatively low additions. In the following sections, the high-dimensional data were first analyzed by PCA to assess overall variances in the data. An RF classification model was then used to validate whether syrup additives can be detected qualitatively, and an RF regression model was used to estimate whether quantitative determination is feasible.

### 3.1. PCA

**Figure 1:**
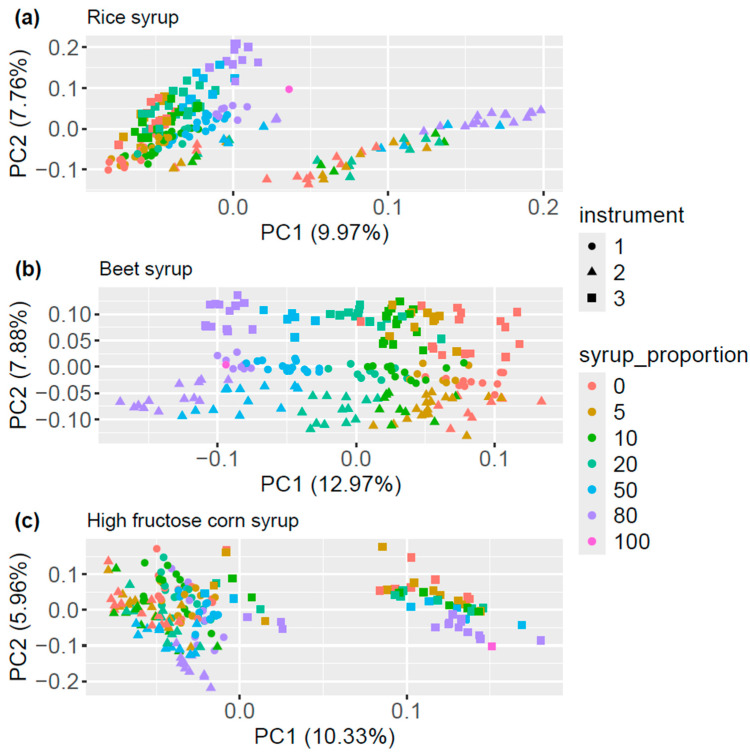
PCA of the fused data of the honey samples adulterated with rice (**a**), beet (**b**) and high-fructose corn syrup (**c**) labeled according to the proportion of syrup [%] (colors) and the device used (shapes).

Figure 1 shows the scores plots of the first two principal components of the analysis of the fused data set of honey samples adulterated with rice (a), beet (b) and high-fructose corn syrup (c). Groupings based on the syrup content can be observed in all three plots. However, an extremely strong influence of the device used is evident, particularly in the scores of the first principal component for the samples adulterated with rice and high-fructose corn syrup and the second principal component for the beet syrup adulteration. Similar results were also obtained when the HILIC and RP data were analyzed individually or when fragment data were added (Figures S1–S5). From this it can be concluded that even after the application of the BOULS data processing approach, device-specific variations were still present in the data to a significant extent. The superposition of these variations with sample-specific variations, which result in a lack of robustness of LC-MS approaches, is generally a well-known challenge when implementing untargeted LC-MS methods in routine laboratories [39,64,65,66]. Therefore, as explained above, LC-MS is currently mainly used either targeted or on a single day by a single device. The processing approach used here nevertheless results in a robust data structure due to the three-dimensional bucketing, which makes it possible to analyze and compare samples over a longer period of time, independent of the processing batch [47].

In the following sections, we will investigate whether a supervised multivariate approach can be used to train models that are based only on the relevant variances and allow device-specific information to be ignored in the evaluation of the authenticity of honey.

### 3.2. Identification of Honey Samples Containing Syrup

To analyze whether honey samples adulterated with syrups can be identified using a supervised multivariate approach, three random forest models were trained to discriminate between the class of pure honey samples and an adulterated class, which included all samples with the addition of one of the syrups. The results for the fused training and test data are shown in Table S1 and Table 1. It can be seen that all pure and beet syrup adulterated samples are correctly classified by the respective model, while one of the test samples adulterated with 10% beet syrup is classified incorrectly. The same classification results for the identification of beet and rice syrup are obtained when the HILIC and RP data are analyzed individually (Tables S2 and S3) and/or fragment data are added (Tables S4–S6). Since the same sample with 10% beet syrup adulteration is misclassified in all data sets but all samples with 5% adulteration are correctly classified, it is likely that the misclassification is due to a pipetting error. The results show that the addition of rice and beet syrup to honey can be clearly detected using LC-MS in combination with BOULS and the application of random forest, a supervised classification approach. For the analysis of high-fructose corn syrup, however, one pure honey sample of the test data (see Table 1) and various samples of the training data (see Table S4) are falsely classified as adulterated, which is also reflected in an increased OOB error of 9.7% for the respective model. This suggests that it is generally more difficult to distinguish between pure honey samples and those adulterated with high-fructose corn syrup than with the other two syrups. This is also supported by the fact that the results vary when analyzing the different data types, so that a larger number of samples are incorrectly assigned when using the pure HILIC data (Table S2), as well as when fragmented data are added (Table S5), than when analyzing the individual data or fused data (Tables S1, S3, S4 and S6). The reason for this is probably that high-fructose corn syrup is more similar to the sugars contained in honey. For example, glucose and fructose each make up 30–40% of high-fructose corn syrup and honey sugars [67], whereas these sugars make up only 1–10% of rice [68] and beet syrup [69]. The fact that almost all samples were correctly classified in the test data set indicates that adulterations with high-fructose corn syrup can also be detected if a sufficiently large data set is used for training. Thus, BOULS data processing combined with random forest classification results in focusing the analysis on the relevant syrup-specific, rather than device-specific, variances, as was already the case for the authentication of honey regarding the geographical origin [47]. The fact that an addition of 5% could be detected for all three syrups shows the great potential of this approach, as the analytical techniques currently used are mostly not capable of this [4,5,35]. This is particularly true for the detection of beet and rice syrups, which are currently particularly difficult to detect [4].

### 3.3. Estimation of the Syrup Content in Adulterated Honey Samples

**Figure 2:**
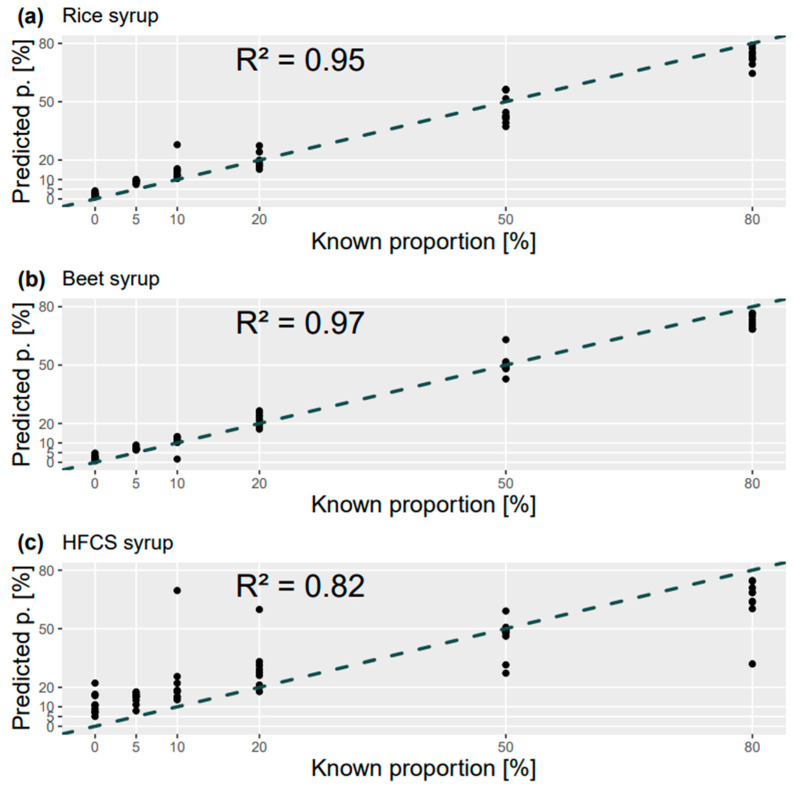
Results of random forest regression showing the predicted proportions of test samples with rice (**a**), beet (**b**) and high-fructose corn syrup (**c**) adulteration plotted against the known proportions.

In order to analyze whether the approach could also be used to predict the syrup content, regression models were trained for each syrup adulteration. Figure 2 shows the results for the analysis of the fused data set. The coefficients of determination show values of 0.95 and 0.97 for the analysis of adulteration with rice and beet syrup, respectively (Figure 2a,b). The fact that these values are quite close to 1 shows that there is a relevant correlation between the respective LC-MS data and the actual proportion of syrup present. This makes it possible to predict the syrup content in the test data with an error that is in the single digits, ranging from 2% to 7% for all analyzed proportions (see Figure 2 and Table S7). Again, the results are quite similar whether the HILIC and RP data are analyzed separately or whether fragment data are added (see Table S7). For the prediction of the high-fructose corn syrup proportion, models with coefficients of determination of around 0.8 are obtained (see Figure 2c and Figure S6–S10). The correlation between the respective LC-MS data and the actual percentage of syrup is therefore much lower here than for the analysis of the other syrups. This is also reflected in the prediction of the test data, which shows larger errors ranging from 8% to 22% for the analysis of all data types (see Figure 2 and Table S7). As already surmised in the previous section, this is probably due to the greater similarity of this syrup to the sugars present in honey. In this case, the device-specific variance present in the data set is probably an obstacle to achieving a more precise determination of the syrup proportion.

Overall, the results show that when analysing LC-MS data of honey samples with BOULS, device-specific information is retained in the data, but its influence can be minimised by analysing the data with a supervised multivariate method. This enables the development of a simple and fast approach, as no re-evaluation of the entire data and no batch correction [70,71], feature identification [72] or feature matching [73] are required for the detection of syrup additives in honey. This can be based on HILIC or RP data or on a fused data set of both approaches for the detection of rice and beet syrup. An addition of fragment data was not necessary here, as no significant improvement in syrup detection was achieved. However, if the approach is used to analyse the addition of a syrup with a honey-like sugar composition, it is possible to identify the addition but it is much more difficult to determine the exact proportion, presumably due to instrumental variations in the data. In this case, it may be useful to combine different mass spectrometric data for a better prediction or even combine LC-MS data with other analytical techniques such as ^13^C-EA/LC-IRMS, which is particularly suitable for C4 sugars like high-fructose corn syrup [4].

## 4. Conclusions and Outlook

We showed that the combination of data processing using BOULS and the application of a supervised multivariate method is very promising for robust and sensitive detection of syrup in honey based on LC-MS, which could be applied in private or state laboratories. In order to establish this approach for routine analysis, the application presented here, a proof-of-concept study using North German honeys, should be further developed for application of samples with higher variance and adulteration with other syrups, e.g., tailored syrups whose compositions are specifically adapted to the composition of the honey that is to be adulterated. In addition, other food samples should also be analyzed, as this approach promises to be robust and sensitive to a wide range of authentication issues.

## Figures and Tables

**Table 1:** Classification results of the fused data of the honey samples adulterated with rice, beet and high-fructose corn syrup. The adulterated class contained the honey samples with 5, 10, 20, 50 and 80% syrup addition. The RF classification model trained with the rice, beet and high-fructose corn syrup data set showed OOB errors of 0%, 0.7% and 9.7%, respectively, and the results of the training data are shown in Table S6.

True Syrup Portion [%]	Correct Predictions Rice Syrup [%]	Correct Predictions Beet Syrup [%]	Correct Predictions High-fructose Corn Syrup [%]
0	9/9	9/9	8/9
5	9/9	9/9	9/9
10	9/9	8/9	9/9
20	9/9	9/9	9/9
50	9/9	9/9	9/9
80	9/9	9/9	9/9

## Data Availability

The data sets analyzed during the current study are not publicly available, as they were generated from samples from customers of a commercial laboratory. However, the BOULS approach is published in an R package here: https://github.com/AGSeifert/BOULS (accessed on 11 November 2024) and example data are provided here: https://www.fdr.uni-hamburg.de/record/13583 (accessed on 11 November 2024).

## Supplementary Materials

The following supporting information can be downloaded at https://www.mdpi.com/article/10.3390/metabo14110633/s1: Figure S1. PCA of the fused data set of the HILIC and RP full-scan and fragment data of the honey samples adulterated with rice (a), beet (b) and high-fructose corn syrup (c) labeled according to the proportion of syrup [%] (colors) and the device used (shapes). Figure S2. PCA of the HILIC full-scan data of the honey samples adulterated with rice (a), beet (b) and high-fructose corn syrup (c) labeled according to the proportion of syrup [%] (colors) and the device used (shapes). Figure S3. PCA of the RP full-scan data of the honey samples adulterated with rice (a), beet (b) and high-fructose corn syrup (c) labeled according to the proportion of syrup [%] (colors) and the device used (shapes). Figure S4. PCA of the HILIC full-scan and fragment data of the honey samples adulterated with rice (a), beet (b) and high-fructose corn syrup (c) labeled according to the proportion of syrup [%] (colors) and the device used (shapes). Figure S5. PCA of the RP full-scan and fragment data of the honey samples adulterated with rice (a), beet (b) and high-fructose corn syrup (c) labeled according to the proportion of syrup [%] (colors) and the device used (shapes). Figure S6. Results of random forest regression for fused full-scan and fragment data showing the predicted proportions of test samples with rice (a), beet (b) and high-fructose corn syrup (c) adulteration plotted against the known proportions. Figure S7. Results of random forest regression for HILIC full-scan data showing the predicted proportions of test samples with rice (a), beet (b) and high-fructose corn syrup (c) adulteration plotted against the known proportions. Figure S8. Results of random forest regression for RP full-scan data showing the predicted proportions of test samples with rice (a), beet (b) and high-fructose corn syrup (c) adulteration plotted against the known proportions. Figure S9. Results of random forest regression for HILIC full-scan and fragment data showing the predicted proportions of test samples with rice (a), beet (b) and high-fructose corn syrup (c) adulteration plotted against the known proportions. Figure S10. Results of random forest regression for RP full-scan and fragment data showing the predicted proportions of test samples with rice (a), beet (b) and high-fructose corn syrup (c) adulteration plotted against the known proportions. Table S1. Classification and regression results of the analysis of the fused full-scan and fragment data of the honey samples adulterated with rice, beet and high-fructose corn syrup. For the classification, the adulterated class contained all of the samples with 5, 10, 20, 50 and 80% addition of the respective syrup (p = pure; a = adulterated; HFCS = high-fructose corn syrup). Table S2. Classification and regression results of the analysis of the fused full-scan data of the honey samples adulterated with rice, beet and high-fructose corn syrup. For the classification, the adulterated class contained all of the samples with 5, 10, 20, 50 and 80% addition of the respective syrup (p = pure; a = adulterated; HFCS = high-fructose corn syrup). Table S3. Classification and regression results of the analysis of the HILIC full-scan data of the honey samples adulterated with rice, beet and high-fructose corn syrup. For the classification, the adulterated class contained all of the samples with 5, 10, 20, 50 and 80% addition of the respective syrup (p = pure; a = adulterated; HFCS = high-fructose corn syrup). Table S4. Classification and regression results of the analysis of the RP full-scan data of the honey samples adulterated with rice, beet and high-fructose corn syrup. For the classification, the adulterated class contained all of the samples with 5, 10, 20, 50 and 80% addition of the respective syrup (p = pure; a = adulterated; HFCS = high-fructose corn syrup). Table S5. Classification and regression results of the analysis of the HILIC full-scan and fragment data of the honey samples adulterated with rice, beet and high-fructose corn syrups. For the classification, the adulterated class contained all of the samples with 5, 10, 20, 50 and 80% addition of the respective syrup (p = pure; a = adulterated). Table S6. Classification and regression results of the analysis of the RP full-scan and fragment data of the honey samples adulterated with rice, beet and high-fructose corn syrup. For the classification, the adulterated class contained all of the samples with 5, 10, 20, 50 and 80% addition of the respective syrup (a = adulterated; p = pure; HFCS = high-fructose corn syrup). Table S7. RMSE for the prediction of the proportions of rice, beet and high-fructose corn syrup in honey samples by the random forest regression models.
