# Supplementary material for: Detection of Sugar Syrups in Honey Using Untargeted Liquid Chromatography–Mass Spectrometry and Chemometrics
Source: Metabolites. 2024 Nov 16;14(11):633. doi: 10.3390/metabo14110633 (PMC11596609; doi:10.3390/metabo14110633)
Supplement: Supplementary file 1 [file metabolites-14-00633-s001.zip › metabolites-3310283-supplementary.pdf]

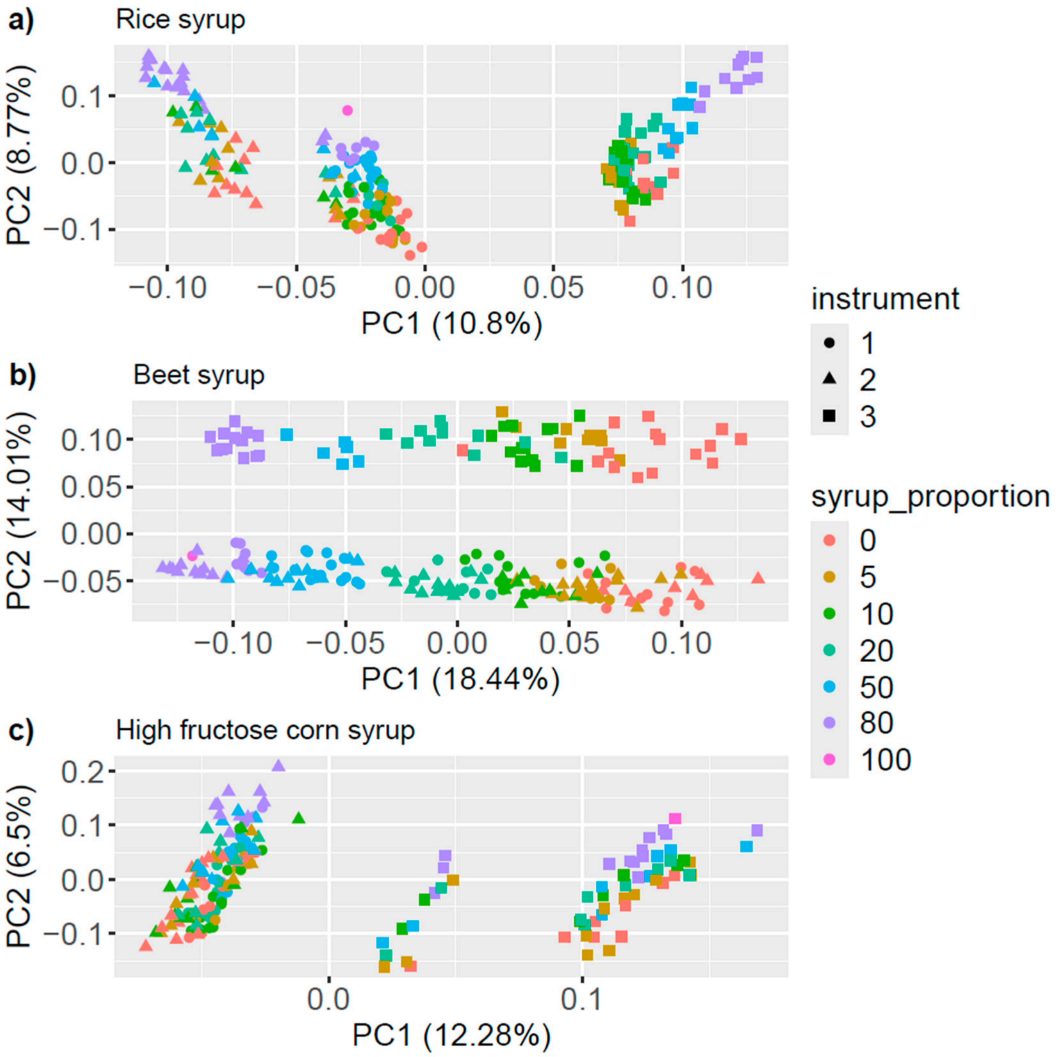

Figure S1. PCA of the fused dataset of the HILIC and RP full scan and fragment data of the honey samples adulterated with rice (a), beet (b) and high fructose corn syrup (c) labeled according to the proportion of syrup [%] (colors) and the device used (shapes).

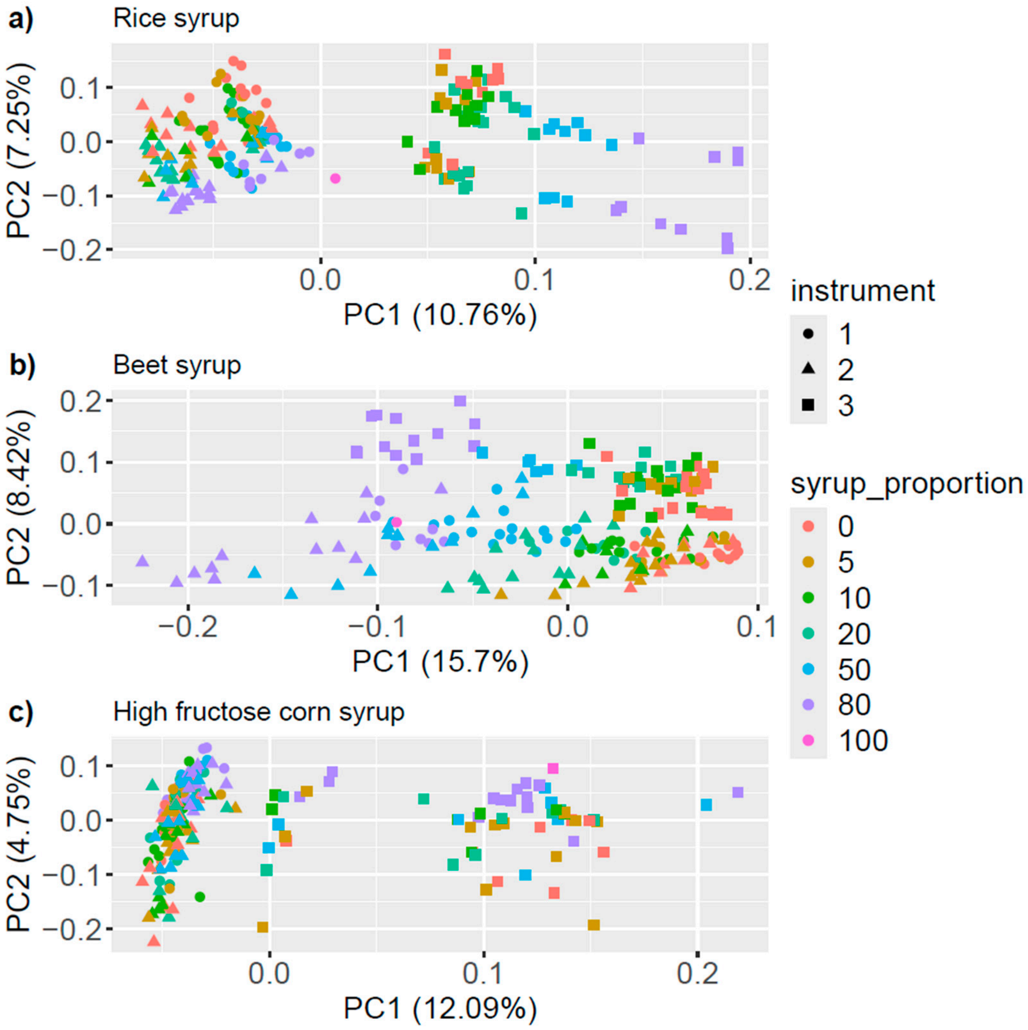

Figure S2. PCA of the HILIC full scan data of the honey samples adulterated with rice (a), beet (b) and high fructose corn syrup (c) labeled according to the proportion of syrup [%] (colors) and the device used (shapes).

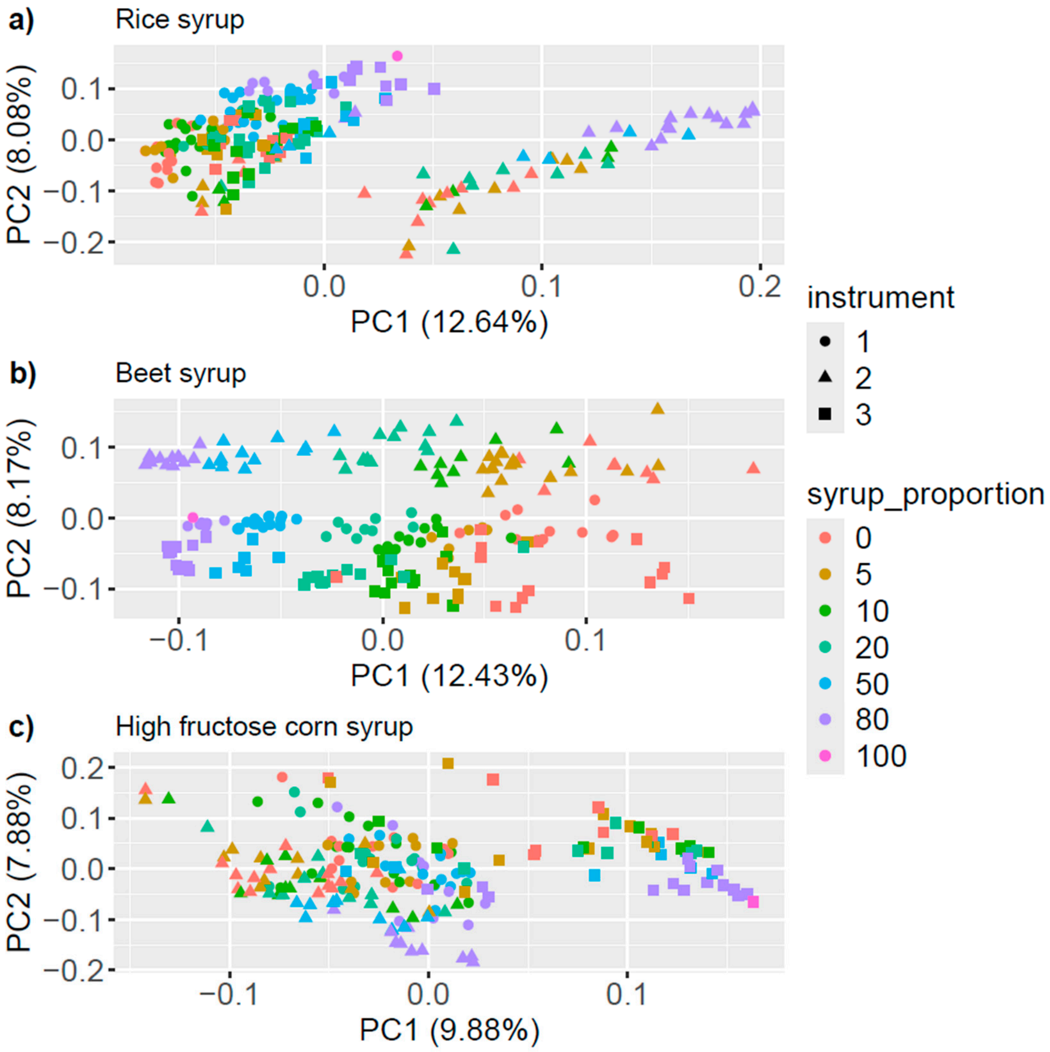

Figure S3. PCA of the RP full scan data of the honey samples adulterated with rice (a), beet (b) and high fructose corn syrup (c) labeled according to the proportion of syrup [%] (colors) and the device used (shapes).

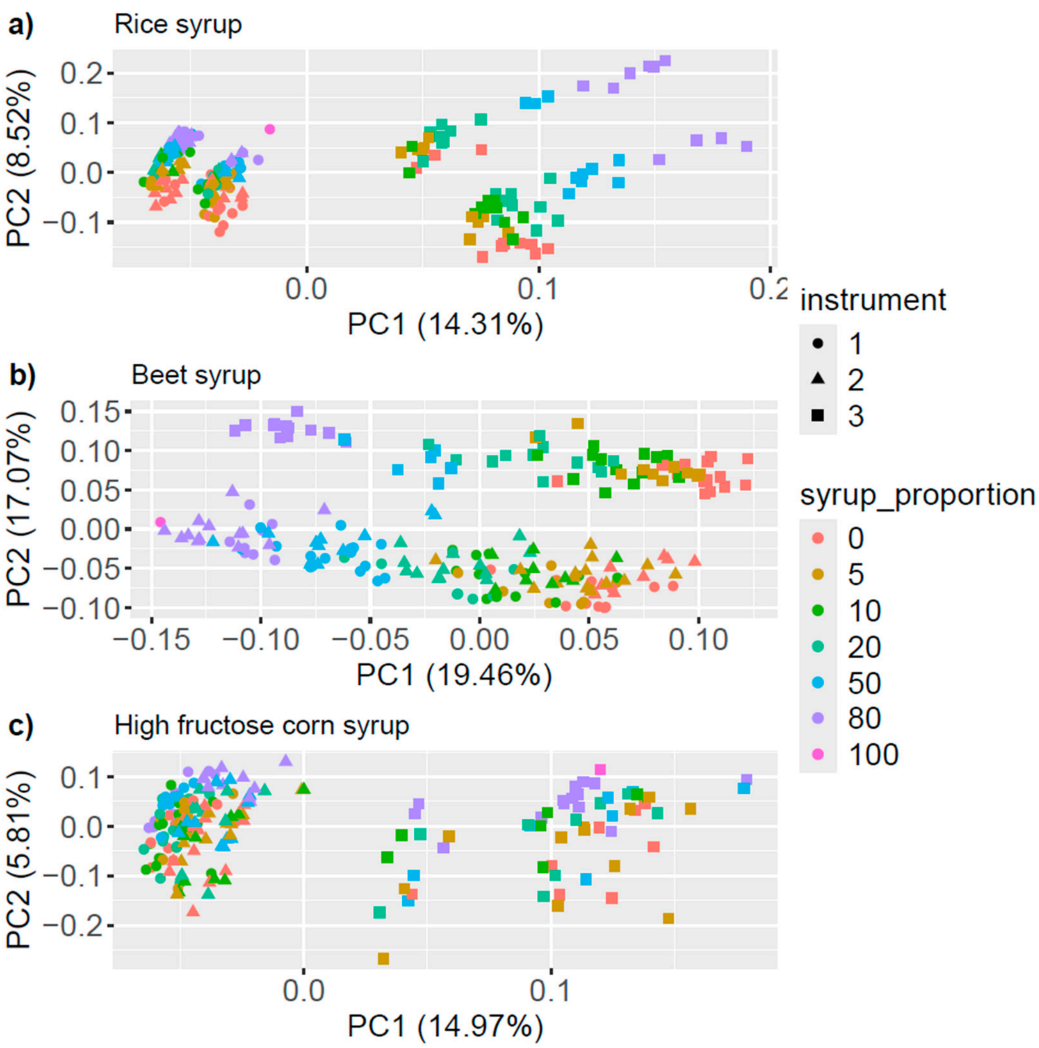

Figure S4. PCA of the HILIC full scan and fragment data of the honey samples adulterated with rice (a), beet (b) and high fructose corn syrup (c) labeled according to the proportion of syrup [%] (colors) and the device used (shapes).

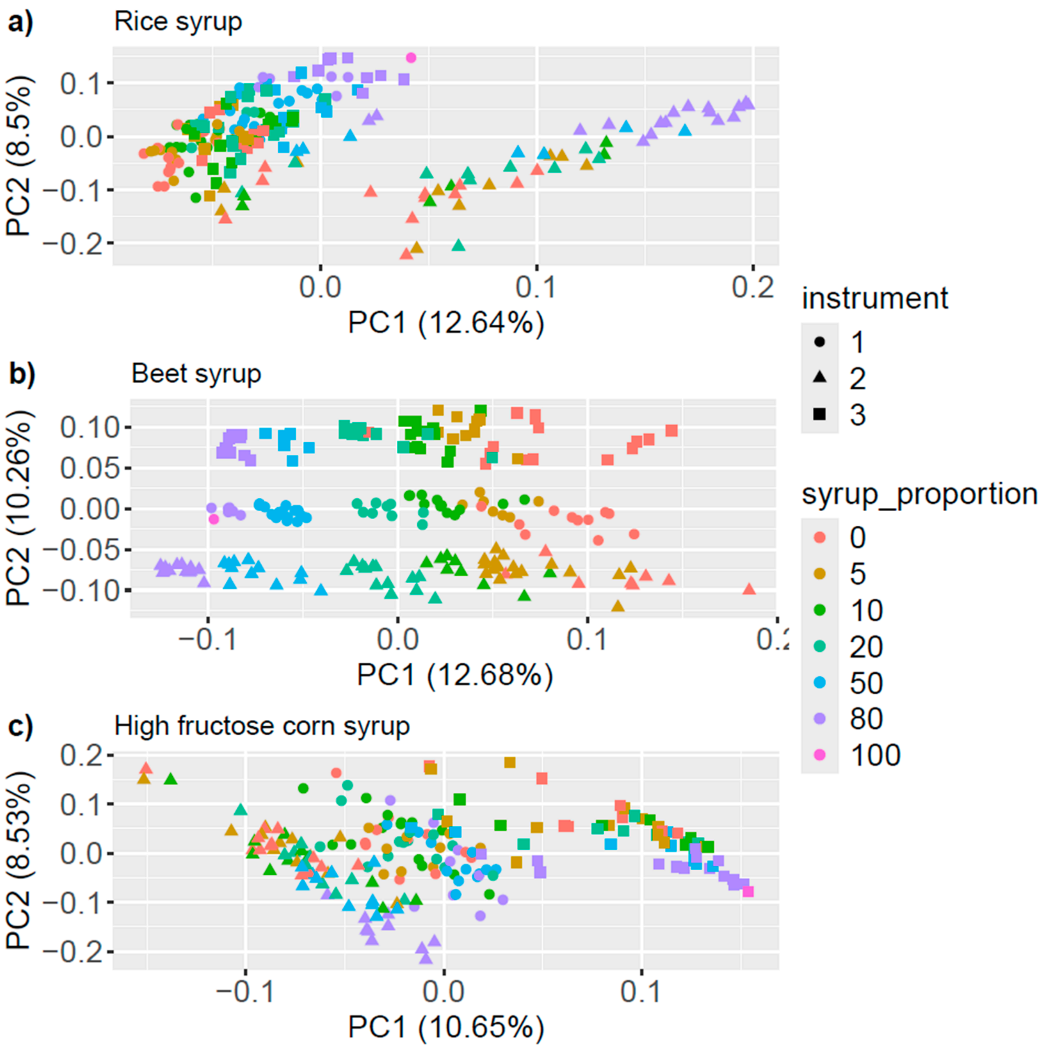

Figure S5. PCA of the RP full scan and fragment data of the honey samples adulterated with rice (a), beet (b) and high fructose corn syrup (c) labeled according to the proportion of syrup [%] (colors) and the device used (shapes).

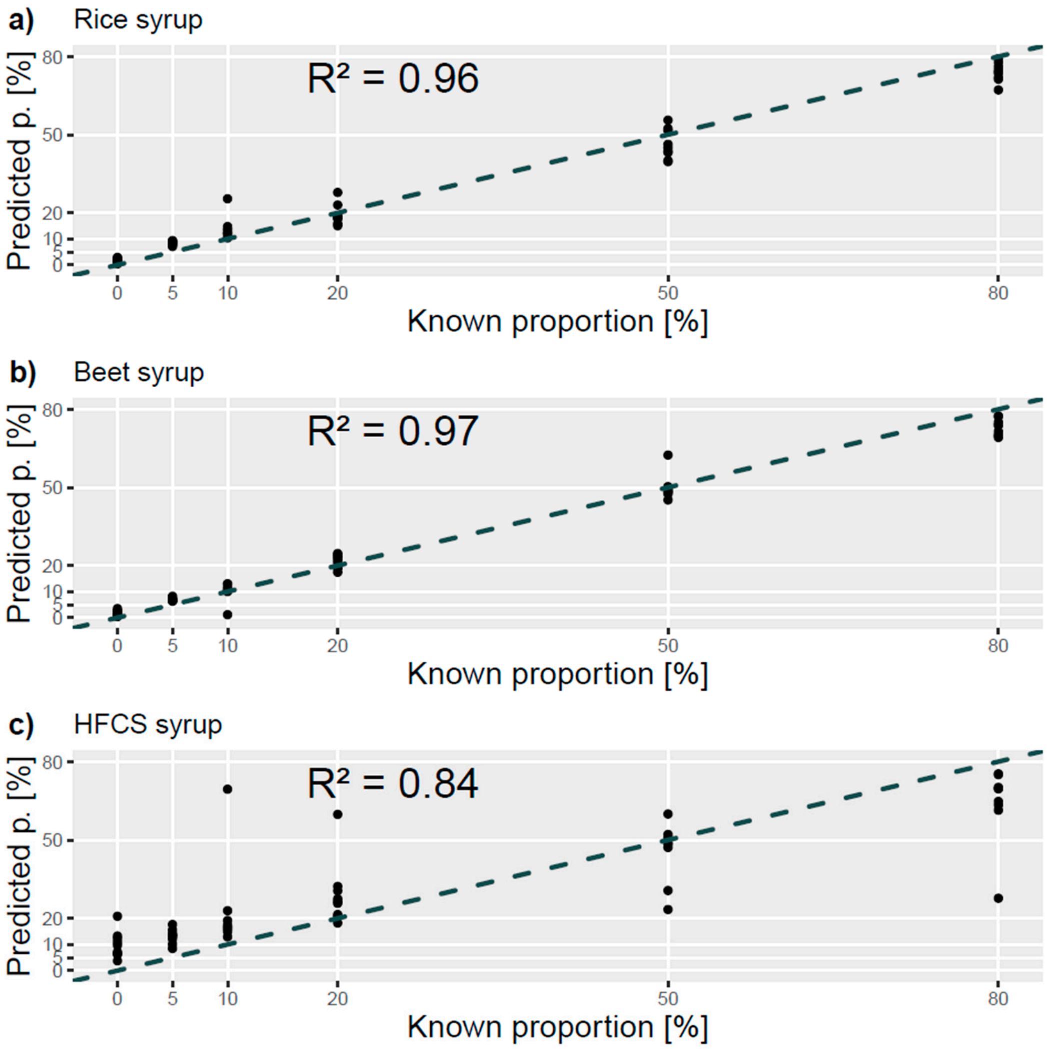

Figure S6. Results of random forest regression for fused full scan and fragment data showing the predicted proportions of test samples with rice (a), beet (b) and high fructose corn syrup (c) adulteration plotted against the known proportions.

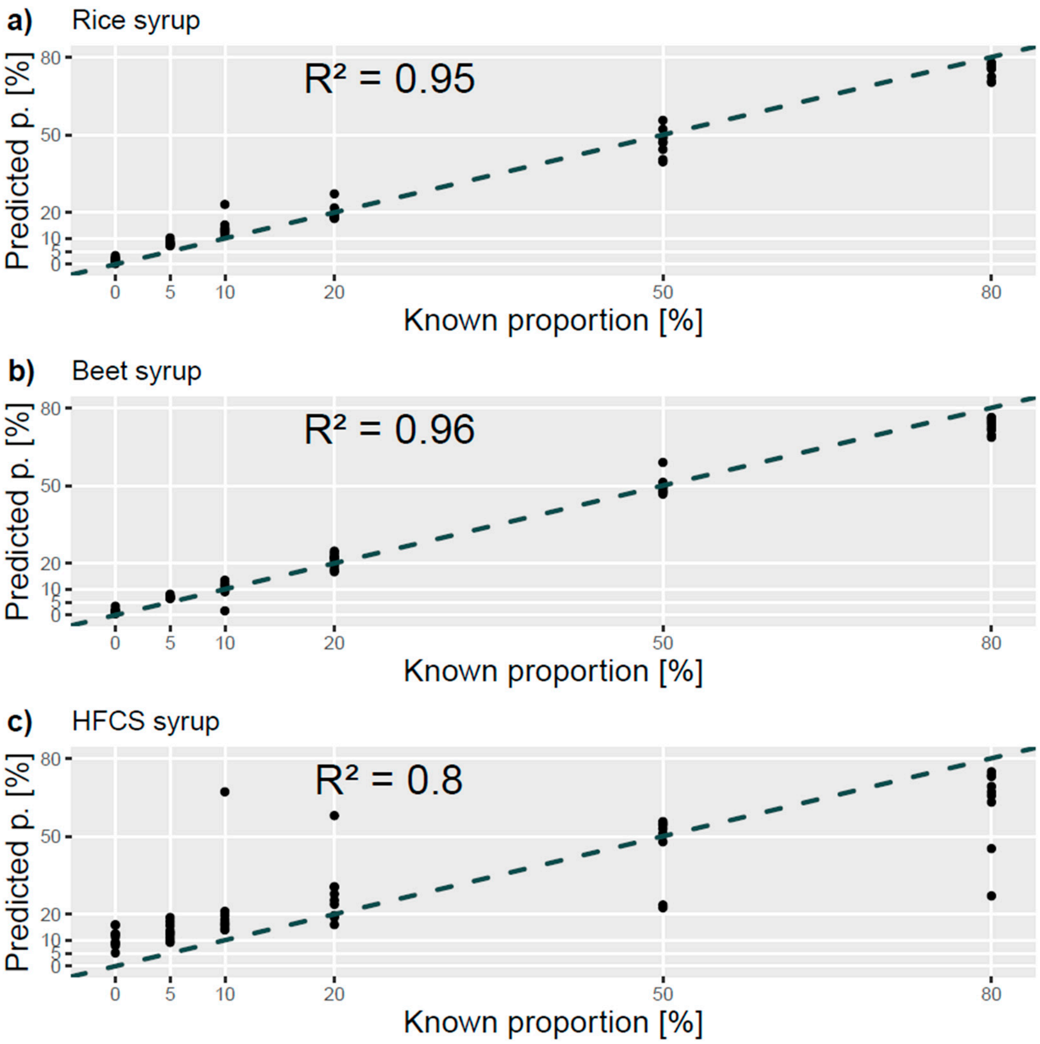

Figure S7. Results of random forest regression for HILIC full scan data showing the predicted proportions of test samples with rice (a), beet (b) and high fructose corn syrup (c) adulteration plotted against the known proportions.

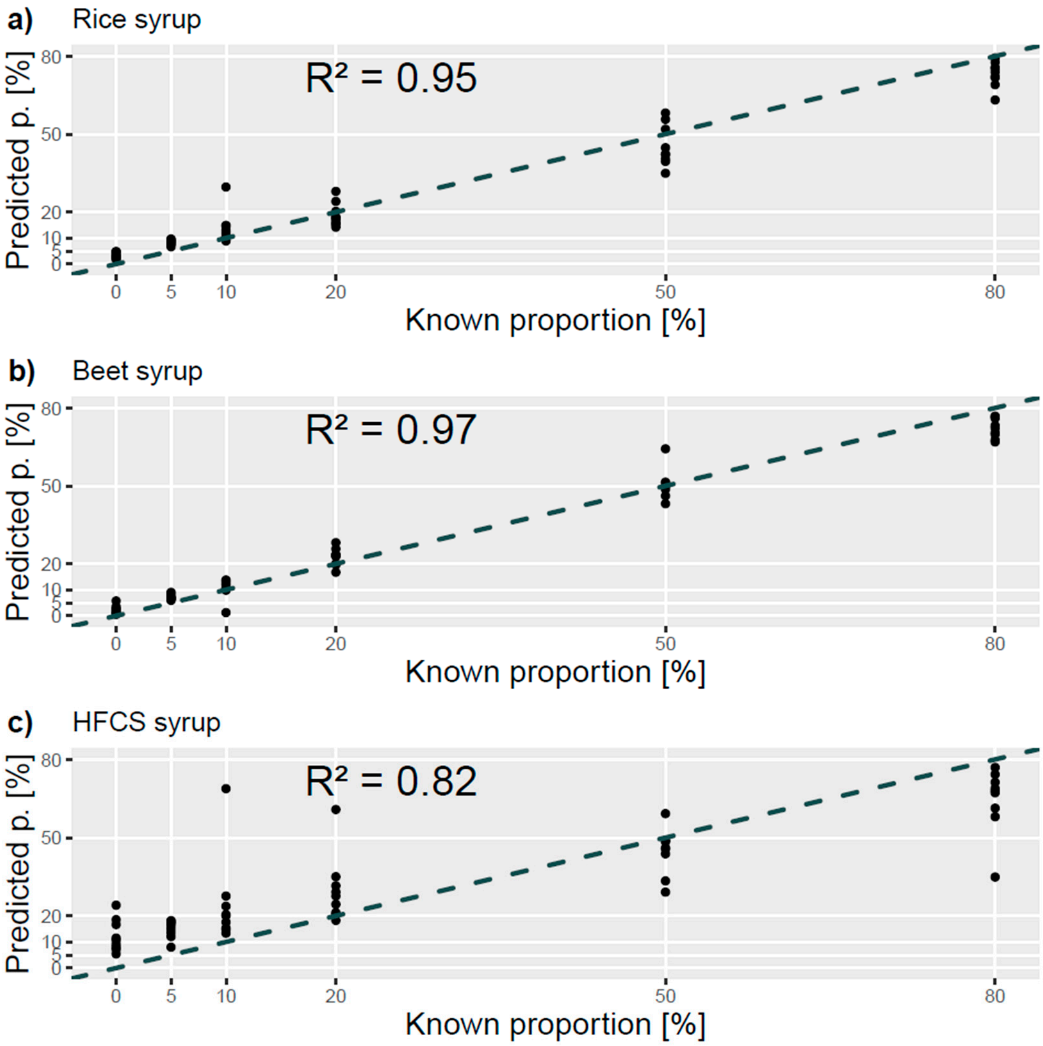

Figure S8. Results of random forest regression for RP full scan data showing the predicted proportions of test samples with rice (a), beet (b) and high fructose corn syrup (c) adulteration plotted against the known proportions.

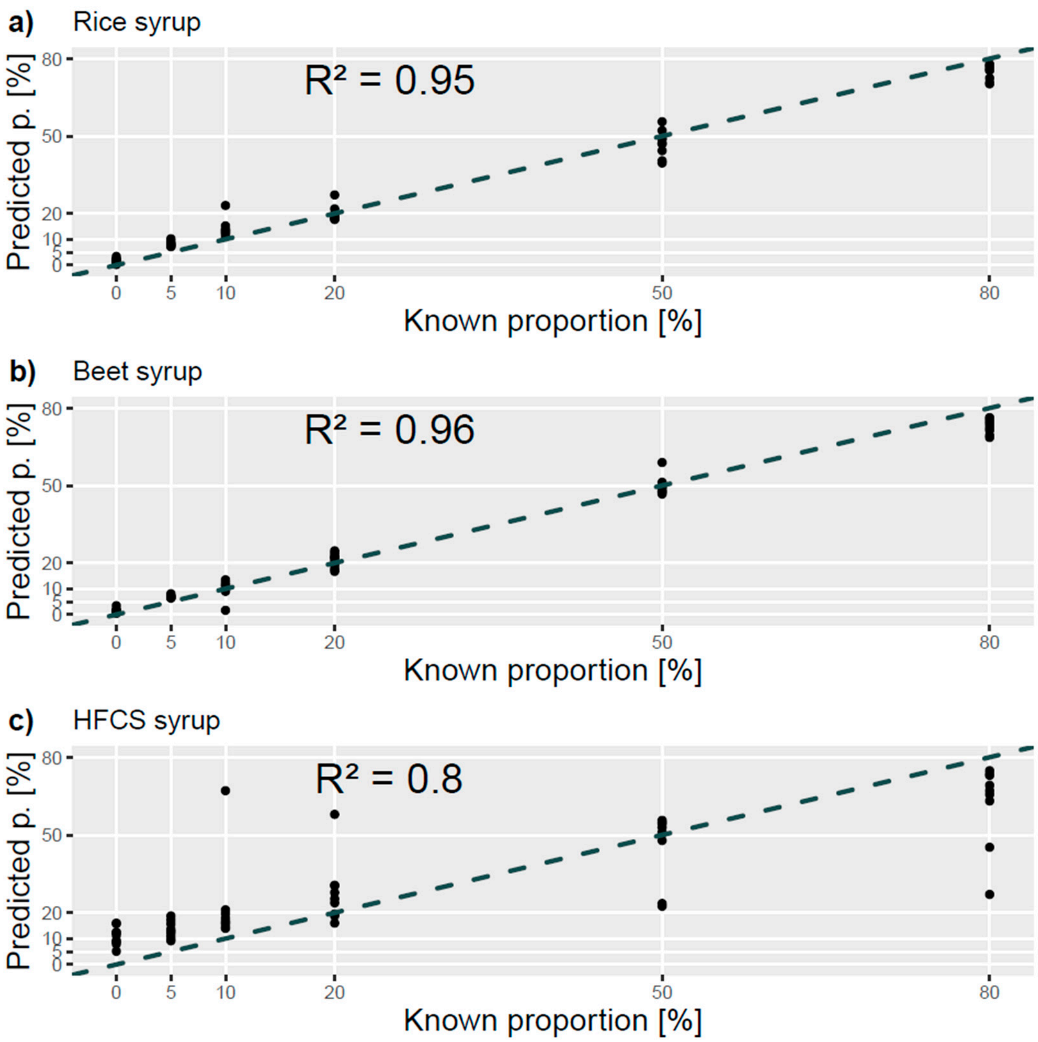

Figure S9. Results of random forest regression for HILIC full scan and fragment data showing the predicted proportions of test samples with rice (a), beet (b) and high fructose corn syrup (c) adulteration plotted against the known proportions.

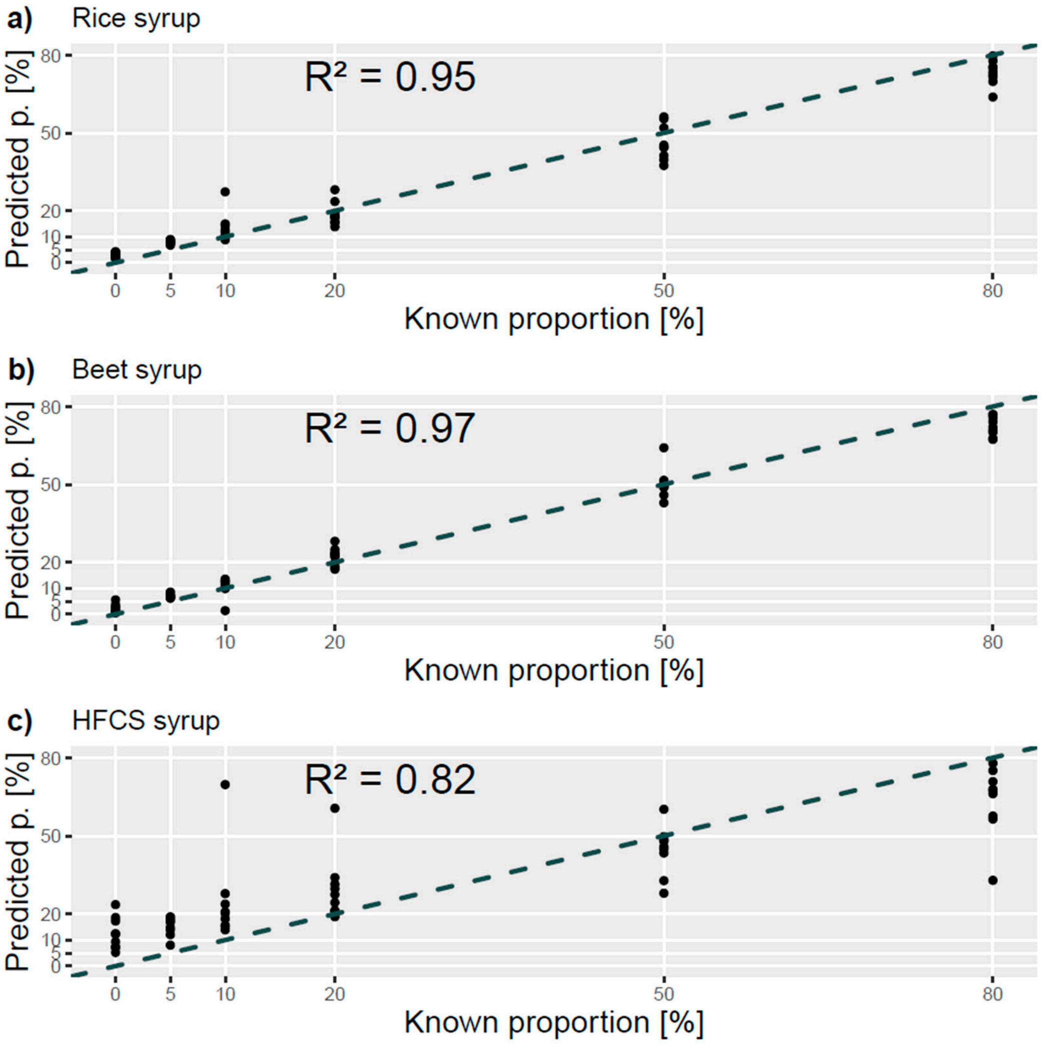

Figure S10. Results of random forest regression for RP full scan and fragment data showing the predicted proportions of test samples with rice (a), beet (b) and high fructose corn syrup (c) adulteration plotted against the known proportions.

Table S1. Classification and regression results of the analysis of the fused full scan data of the honey samples adulterated with rice, beet and high fructose corn syrup. For the classification, the adulterated class contained all of the samples with 5, 10, 20, 50 and 80% addition of the respective syrup (p = pure, a = adulterated, HFCS = high fructose corn syrup).

Honey no.	Dataset	True syrup proportion [%]	Classification rice syrup	Proportion estimation rice syrup [%]	Classification beet syrup	Proportion estimation beet syrup [%]	Classification HFCS	Proportion estimation HFCS [%]
1	test	0	p	2.02	p	3.42	a	22.2
2	test	0	p	1.64	p	2.33	p	15.7
3	test	0	p	1.79	p	1.37	p	10.6
4	test	0	p	1.04	p	1.24	p	7.57
5	test	0	p	3.84	p	0.90	p	6.99
6	test	0	p	2.90	p	2.74	p	11.1
7	test	0	p	3.30	p	4.76	p	16.5
8	test	0	p	2.23	p	0.72	p	5.05
9	test	0	p	0.93	p	0.99	p	8.92
10	training	0	p	1.55	p	0.77	a	10.29
11	training	0	p	1.06	p	1.78	a	7.46
12	training	0	p	3.12	p	0.85	a	8.43
13	training	0	p	1.85	p	1.81	p	6.41
14	training	0	p	1.30	p	0.75	p	8.13
15	training	0	p	1.27	p	0.61	p	6.18
16	training	0	p	1.40	p	1.56	p	8.65
17	training	0	p	2.38	p	1.95	a	13.21
18	training	0	p	3.89	p	2.49	a	9.22
19	training	0	p	3.64	p	2.58	a	12.88
20	training	0	p	1.02	p	0.98	p	6.40
21	training	0	p	1.44	p	0.91	a	14.73
22	training	0	p	0.78	p	1.32	p	6.09
23	training	0	p	2.56	p	0.89	p	5.50
24	training	0	p	3.69	p	0.74	p	5.03
25	training	0	p	1.00	p	1.89	p	5.58
26	training	0	p	2.55	p	1.12	p	6.51
27	training	0	p	1.58	p	1.27	a	6.39
28	training	0	p	1.77	p	1.28	a	10.37
29	training	0	p	2.01	p	4.89	a	13.59
30	training	0	p	3.11	p	1.70	a	7.42
31	training	0	p	1.53	p	1.56	a	11.37
32	training	0	p	1.18	a	19.3	a	7.54
33	training	0	p	1.80	p	1.60	a	6.82
34	training	0	p	1.49	p	2.06	a	12.5
35	test	5	a	10.0	a	8.04	a	15.5
36	test	5	a	8.58	a	6.91	a	17.7
37	test	5	a	9.08	a	6.76	a	16.8
38	test	5	a	7.44	a	6.32	a	7.93
39	test	5	a	9.26	a	7.12	a	15.2
40	test	5	a	9.37	a	6.91	a	15.1
41	test	5	a	9.04	a	8.92	a	16.2
42	test	5	a	8.34	a	6.70	a	13.4

43	test	5	a	8.19	a	6.63	a	11.1
44	training	5	a	8.56	a	7.11	a	10.3
45	training	5	a	7.31	a	6.01	a	13.1
46	training	5	a	8.24	a	6.16	a	9.63
47	training	5	a	8.08	a	7.20	a	15.2
48	training	5	a	7.94	a	6.89	a	13.8
49	training	5	a	7.78	a	6.29	a	16.9
50	training	5	a	9.40	a	6.36	a	15.3
51	training	5	a	10.4	a	8.01	a	10.7
52	training	5	a	9.26	a	7.45	a	10.0
53	training	5	a	11.5	a	6.05	a	7.29
54	training	5	a	11.2	a	7.75	a	8.04
55	training	5	a	7.98	a	10.2	a	12.4
56	training	5	a	9.37	a	6.61	a	18.2
57	training	5	a	10.6	a	6.90	a	7.78
58	training	5	a	9.99	a	7.41	a	13.7
59	training	5	a	10.3	a	6.43	a	14.5
60	training	5	a	10.7	a	10.3	a	39.0
61	training	5	a	9.11	a	7.39	a	8.79
62	training	5	a	7.35	a	6.43	a	7.98
63	training	5	a	8.06	a	8.32	a	10.8
64	training	5	a	9.07	a	6.64	a	9.23
65	training	5	a	8.70	a	6.13	a	8.48
66	training	5	a	8.46	a	7.06	a	9.08
67	training	5	a	8.85	a	7.77	a	11.5
68	training	5	a	8.98	a	6.26	a	15.9
69	test	10	a	12.4	a	13.2	a	17.7
70	test	10	a	10.4	a	10.5	a	25.6
71	test	10	a	12.1	a	11.5	a	15.3
72	test	10	a	10.6	a	11.4	a	13.6
73	test	10	a	28.6	a	13.2	a	14.7
74	test	10	a	15.6	p	1.68	a	18.5
75	test	10	a	11.8	a	12.4	a	22.1
76	test	10	a	14.7	a	10.3	a	18.2
77	test	10	a	13.5	a	10.3	a	69.5
78	training	10	a	9.38	a	9.28	a	22.8
79	training	10	a	9.84	a	10.8	a	21.5
80	training	10	a	15.7	a	12.8	a	18.9
81	training	10	a	24.2	a	10.8	a	18.6
82	training	10	a	23.8	a	10.9	a	12.9
83	training	10	a	13.3	a	10.8	a	12.2
84	training	10	a	10.6	a	14.7	a	8.77
85	training	10	a	15.7	a	8.19	a	18.4
86	training	10	a	7.55	a	11.4	a	15.1
87	training	10	a	16.5	a	10.4	a	16.4
88	training	10	a	11.7	a	12.3	a	12.6
89	training	10	a	10.7	a	13.4	a	11.7
90	training	10	a	11.9	a	11.9	a	9.98
91	training	10	a	11.5	a	13.8	a	15.2
92	training	10	a	12.5	a	12.1	a	11.2
93	training	10	a	11.7	a	10.8	a	10.5

94	training	10	a	11.6	a	10.3	a	19.4
95	training	10	a	12.3	a	10.1	a	14.2
96	training	10	a	9.52	a	9.84	a	12.6
97	training	10	a	9.96	a	11.7	a	10.4
98	training	10	a	11.8	a	10.4	a	12.1
99	training	10	a	13.0	a	12.1	a	13.1
100	training	10	a	9.94	a	12.1	a	13.3
101	training	10	a	11.0	a	10.9	a	10.1
102	training	10	a	13.2	a	11.8	a	9.86
103	test	20	a	17.3	a	18.5	a	27.6
104	test	20	a	15.0	a	19.8	a	59.8
105	test	20	a	19.7	a	24.0	a	31.3
106	test	20	a	18.2	a	26.5	a	26.1
107	test	20	a	27.8	a	23.8	a	33.1
108	test	20	a	17.6	a	17.1	a	17.8
109	test	20	a	18.4	a	22.1	a	29.1
110	test	20	a	23.6	a	21.8	a	20.2
111	test	20	a	16.3	a	25.6	a	21.2
112	training	20	a	17.9	a	17.2	a	49.1
113	training	20	a	17.8	a	16.9	a	18.9
114	training	20	a	29.5	a	17.7	a	17.3
115	training	20	a	40.5	a	23.6	a	18.4
116	training	20	a	23.8	a	24.8	a	29.6
117	training	20	a	20.1	a	21.5	a	23.4
118	training	20	a	19.2	a	18.7	a	28.8
119	training	20	a	27.9	a	20.7	a	17.0
120	training	20	a	14.7	a	20.2	a	25.8
121	training	20	a	17.6	a	24.7	a	14.6
122	training	20	a	41.5	a	25.4	a	23.2
123	training	20	a	18.0	a	25.0	a	21.8
124	training	20	a	24.6	a	24.7	a	34.6
125	training	20	a	17.8	a	23.6	a	24.5
126	training	20	a	20.0	a	18.5	a	28.2
127	training	20	a	15.1	a	15.6	a	16.5
128	training	20	a	17.1	a	16.7	a	22.7
129	training	20	a	22.9	a	17.8	a	16.3
130	training	20	a	18.2	a	20.5	a	17.7
131	training	20	a	17.9	a	22.1	a	19.4
132	training	20	a	13.9	a	25.1	a	19.2
133	training	20	a	16.7	a	20.4	a	16.3
134	training	20	a	18.6	a	24.0	a	21.6
135	training	20	a	22.5	a	19.8	a	20.3
136	training	20	a	29.8	a	8.3	a	21.4
137	test	50	a	43.4	a	48.1	a	48.4
138	test	50	a	42.3	a	48.7	a	47.7
139	test	50	a	51.7	a	48.5	a	27.3
140	test	50	a	39.3	a	50.1	a	49.7
141	test	50	a	56.3	a	63.1	a	50.7
142	test	50	a	41.8	a	50.4	a	31.5
143	test	50	a	37.3	a	42.9	a	46.1
144	test	50	a	56.4	a	50.1	a	59.1

145	test	50	a	45.2	a	51.7	a	48.9
146	training	50	a	42.5	a	57.4	a	64.2
147	training	50	a	59.5	a	43.0	a	53.9
148	training	50	a	50.3	a	46.8	a	53.3
149	training	50	a	51.8	a	48.3	a	64.7
150	training	50	a	54.6	a	54.1	a	47.9
151	training	50	a	56.9	a	49.7	a	49.4
152	training	50	a	42.1	a	52.3	a	42.9
153	training	50	a	41.0	a	52.3	a	44.8
154	training	50	a	53.1	a	59.0	a	42.9
155	training	50	a	35.0	a	59.8	a	45.9
156	training	50	a	52.1	a	51.7	a	39.1
157	training	50	a	47.6	a	55.5	a	54.2
158	training	50	a	54.9	a	41.0	a	50.9
159	training	50	a	38.4	a	53.2	a	59.8
160	training	50	a	56.5	a	54.9	a	43.4
161	training	50	a	54.6	a	43.7	a	46.7
162	training	50	a	46.4	a	49.1	a	44.7
163	training	50	a	39.3	a	50.1	a	18.1
164	training	50	a	34.5	a	59.9	a	39.7
165	training	50	a	39.7	a	54.4	a	34.3
166	training	50	a	37.9	a	51.0	a	46.6
167	training	50	a	48.5	a	53.0	a	51.6
168	training	50	a	40.5	a	52.0	a	51.9
169	training	50	a	46.2	a	57.8	a	64.2
170	training	50	a	61.2	a	53.1	a	44.9
171	test	80	a	72.5	a	68.4	a	68.5
172	test	80	a	64.8	a	75.1	a	68.5
173	test	80	a	77.5	a	71.7	a	74.7
174	test	80	a	75.9	a	68.6	a	64.0
175	test	80	a	79.0	a	76.7	a	74.3
176	test	80	a	74.6	a	68.8	a	63.6
177	test	80	a	69.7	a	70.4	a	60.2
178	test	80	a	73.3	a	73.3	a	32.0
179	test	80	a	71.8	a	76.4	a	70.9
180	training	80	a	75.6	a	70.6	a	25.5
181	training	80	a	73.8	a	69.0	a	31.2
182	training	80	a	79.2	a	71.4	a	68.5
183	training	80	a	78.3	a	68.6	a	68.1
184	training	80	a	78.6	a	69.1	a	12.9
185	training	80	a	78.5	a	73.5	a	60.6
186	training	80	a	74.2	a	71.1	a	75.7
187	training	80	a	76.0	a	73.5	a	71.5
188	training	80	a	67.5	a	71.4	a	54.6
189	training	80	a	75.3	a	74.7	a	75.1
190	training	80	a	74.4	a	73.6	a	68.3
191	training	80	a	76.8	a	65.2	a	73.9
192	training	80	a	75.1	a	70.4	a	73.4
193	training	80	a	76.5	a	76.1	a	68.4
194	training	80	a	76.3	a	72.5	a	72.2
195	training	80	a	72.1	a	77.3	a	65.0

196	training	80	a	65.4	a	75.6	a	63.4
197	training	80	a	72.4	a	78.6	a	68.7
198	training	80	a	63.9	a	78.5	a	72.1
199	training	80	a	67.4	a	75.2	a	73.9
200	training	80	a	66.6	a	76.0	a	75.6
201	training	80	a	72.9	a	76.6	a	78.5
202	training	80	a	68.2	a	76.6	a	68.7
203	training	80	a	73.8	a	77.3	a	66.3
204	training	80	a	79.0	a	74.1	a	77.0
205	training	100	a	78.1	a	69.2	a	61.8

Table S2. Classification and regression results of the analysis of the HILIC full scan data of the honey samples adulterated with rice, beet and high fructose corn syrup. For the classification, the adulterated class contained all of the samples with 5, 10, 20, 50 and 80% addition of the respective syrup (p = pure, a = adulterated, HFCS = high fructose corn syrup).

Honey no.	Dataset	True syrup proportion [%]	Classification rice syrup	Proportion estimation rice syrup [%]	Classification beet syrup	Proportion estimation beet syrup [%]	Classification HFCS	Proportion estimation HFCS [%]
1	test	0	p	2.22	p	4.07	a	15.7
2	test	0	p	2.11	p	3.20	a	15.4
3	test	0	p	1.52	p	2.29	a	12.4
4	test	0	p	1.23	p	3.46	p	8.19
5	test	0	p	4.02	p	1.56	a	8.52
6	test	0	p	3.47	p	5.76	a	11.7
7	test	0	p	5.09	p	4.82	a	12.4
8	test	0	p	3.46	p	1.32	p	5.27
9	test	0	p	1.00	p	2.05	a	9.09
10	training	0	p	4.27	p	1.06	a	12.2
11	training	0	p	2.06	p	3.35	a	9.85
12	training	0	p	2.02	p	1.24	a	9.46
13	training	0	p	3.10	p	5.62	p	8.53
14	training	0	p	1.08	p	0.85	a	9.12
15	training	0	p	0.95	p	1.50	p	7.25
16	training	0	p	1.27	p	3.03	p	7.64
17	training	0	p	2.55	p	2.71	a	11.9
18	training	0	p	3.36	p	2.88	a	10.6
19	training	0	p	2.19	p	4.06	a	13.0
20	training	0	p	0.97	p	1.76	a	9.01
21	training	0	p	1.44	p	1.79	a	16.2
22	training	0	p	1.14	p	3.06	p	8.64
23	training	0	p	4.50	p	2.07	p	7.57
24	training	0	p	6.45	p	1.41	p	6.19
25	training	0	p	0.91	p	2.38	a	11.5
26	training	0	p	5.30	p	1.71	p	7.47
27	training	0	p	1.89	p	2.58	p	6.30
28	training	0	p	2.05	p	2.00	a	8.68
29	training	0	p	2.43	p	1.94	a	13.4
30	training	0	p	2.25	p	4.79	a	11.7
31	training	0	p	1.61	p	5.35	a	9.75
32	training	0	p	1.25	a	18.4	a	11.5
33	training	0	p	2.24	p	3.97	a	8.66
34	training	0	p	2.38	p	3.88	a	12.5
35	test	5	a	11.9	a	8.06	a	11.1
36	test	5	a	9.30	a	6.10	a	17.3
37	test	5	a	8.70	a	7.04	a	18.6
38	test	5	a	8.56	a	7.66	a	9.11
39	test	5	a	8.41	a	7.18	a	12.5
40	test	5	a	9.42	a	9.02	p	14.8
41	test	5	a	10.7	a	9.81	a	12.4

42	test	5	a	8.36	a	6.63	a	13.8
43	test	5	a	8.25	a	7.18	a	9.51
44	training	5	a	9.49	a	10.1	a	10.5
45	training	5	a	8.41	a	5.75	a	12.5
46	training	5	a	9.22	a	6.17	a	10.7
47	training	5	a	9.41	a	8.11	a	13.2
48	training	5	a	8.37	a	9.04	a	15.6
49	training	5	a	10.4	a	6.14	a	17.8
50	training	5	a	9.27	a	6.33	a	13.8
51	training	5	a	11.4	a	8.28	a	10.4
52	training	5	a	8.73	a	7.75	a	11.3
53	training	5	a	13.4	a	6.50	a	7.60
54	training	5	a	10.0	a	8.63	a	8.72
55	training	5	a	9.26	a	11.8	a	11.6
56	training	5	a	10.4	a	6.88	a	13.2
57	training	5	a	12.1	a	6.75	a	8.68
58	training	5	a	10.7	a	7.21	a	9.83
59	training	5	a	12.5	a	6.99	a	13.7
60	training	5	a	11.1	a	7.10	a	12.4
61	training	5	a	9.28	a	7.32	a	28.6
62	training	5	a	10.3	a	7.07	a	10.9
63	training	5	a	8.71	a	9.83	a	9.92
64	training	5	a	11.0	a	7.65	a	16.6
65	training	5	a	9.41	a	7.12	a	12.3
66	training	5	a	10.3	a	8.62	a	11.5
67	training	5	a	9.2	a	10.2	a	9.87
68	training	5	a	12.0	a	6.45	a	15.9
69	test	10	a	14.7	a	12.3	a	14.5
70	test	10	a	14.1	a	12.0	a	20.7
71	test	10	a	12.6	a	9.69	a	17.3
72	test	10	a	13.1	a	13.4	a	14.2
73	test	10	a	24.3	a	14.8	a	15.9
74	test	10	a	16.9	p	4.21	a	15.9
75	test	10	a	15.1	a	8.84	a	16.2
76	test	10	a	14.2	a	11.5	a	18.9
77	test	10	a	20.0	a	10.2	a	67.5
78	training	10	a	10.1	a	8.82	a	22.3
79	training	10	a	12.5	a	12.5	a	21.3
80	training	10	a	22.0	a	16.4	a	17.4
81	training	10	a	28.2	a	13.3	a	18.3
82	training	10	a	27.6	a	13.0	a	14.2
83	training	10	a	17.3	a	11.0	a	11.3
84	training	10	a	13.1	a	16.0	a	9.76
85	training	10	a	15.9	a	7.49	a	21.9
86	training	10	a	8.25	a	12.0	a	14.6
87	training	10	a	12.6	a	11.0	a	14.9
88	training	10	a	12.4	a	12.6	a	11.3
89	training	10	a	11.9	a	14.6	a	11.0
90	training	10	a	14.8	a	13.0	a	10.8
91	training	10	a	14.1	a	13.3	a	14.6
92	training	10	a	12.5	a	8.91	a	13.9

93	training	10	a	14.2	a	9.59	a	9.97
94	training	10	a	13.1	a	9.62	a	10.5
95	training	10	a	14.0	a	9.89	a	20.7
96	training	10	a	10.7	a	10.1	a	12.2
97	training	10	a	11.0	a	12.6	a	10.2
98	training	10	a	12.9	a	10.6	a	12.8
99	training	10	a	14.3	a	15.8	a	14.9
100	training	10	a	10.8	a	14.0	a	18.1
101	training	10	a	12.6	a	11.1	a	15.3
102	training	10	a	13.6	a	10.6	a	9.86
103	test	20	a	19.4	a	16.1	a	24.0
104	test	20	a	20.0	a	17.7	a	57.6
105	test	20	a	19.7	a	26.5	a	31.0
106	test	20	a	18.5	a	23.0	a	27.9
107	test	20	a	28.8	a	26.6	a	30.3
108	test	20	a	18.5	a	18.4	a	16.4
109	test	20	a	20.6	a	20.2	a	25.3
110	test	20	a	23.1	a	18.7	a	18.4
111	test	20	a	20.8	a	24.4	a	19.5
112	training	20	a	22.2	a	20.4	a	51.7
113	training	20	a	18.5	a	19.0	a	18.2
114	training	20	a	32.9	a	16.0	a	14.2
115	training	20	a	35.0	a	27.1	a	20.5
116	training	20	a	22.3	a	26.8	a	34.1
117	training	20	a	19.8	a	16.7	a	24.1
118	training	20	a	19.2	a	16.5	a	31.6
119	training	20	a	19.4	a	19.5	a	20.1
120	training	20	a	17.0	a	20.3	a	20.8
121	training	20	a	21.0	a	26.9	a	14.4
122	training	20	a	37.8	a	23.1	a	26.4
123	training	20	a	20.8	a	23.3	a	21.5
124	training	20	a	23.0	a	21.6	a	39.1
125	training	20	a	18.5	a	20.7	a	24.1
126	training	20	a	21.9	a	17.0	a	21.1
127	training	20	a	19.1	a	17.6	a	22.5
128	training	20	a	22.1	a	13.7	a	17.5
129	training	20	a	23.2	a	17.5	a	25.8
130	training	20	a	18.9	a	24.5	a	27.3
131	training	20	a	21.2	a	21.3	a	18.8
132	training	20	a	19.1	a	24.4	a	25.3
133	training	20	a	18.4	a	19.1	a	20.1
134	training	20	a	20.6	a	24.8	a	26.2
135	training	20	a	23.3	a	18.4	a	22.6
136	training	20	a	27.8	a	11.6	a	21.4
137	test	50	a	45.9	a	51.6	a	53.4
138	test	50	a	47.3	a	45.4	a	51.1
139	test	50	a	53.1	a	47.9	a	22.5
140	test	50	a	38.1	a	52.2	a	52.6
141	test	50	a	57.9	a	58.6	a	55.9
142	test	50	a	34.6	a	53.2	a	23.2
143	test	50	a	44.6	a	43.6	a	47.9

144	test	50	a	48.7	a	48.6	a	55.3
145	test	50	a	50.2	a	50.2	a	50.8
146	training	50	a	33.7	a	53.7	a	63.2
147	training	50	a	54.6	a	42.3	a	56.5
148	training	50	a	52.2	a	48.9	a	57.9
149	training	50	a	50.7	a	46.9	a	65.0
150	training	50	a	52.8	a	54.5	a	51.8
151	training	50	a	59.8	a	52.0	a	53.5
152	training	50	a	46.3	a	52.5	a	40.0
153	training	50	a	45.6	a	55.5	a	38.9
154	training	50	a	53.0	a	63.9	a	61.0
155	training	50	a	41.9	a	57.2	a	45.4
156	training	50	a	50.0	a	50.8	a	43.8
157	training	50	a	42.3	a	54.0	a	36.7
158	training	50	a	35.2	a	40.1	a	53.1
159	training	50	a	40.2	a	47.2	a	57.6
160	training	50	a	53.3	a	51.8	a	57.0
161	training	50	a	52.3	a	45.7	a	42.2
162	training	50	a	46.7	a	52.8	a	43.6
163	training	50	a	38.8	a	53.1	a	34.4
164	training	50	a	34.9	a	65.6	a	17.5
165	training	50	a	39.7	a	60.4	a	35.8
166	training	50	a	37.3	a	53.7	a	41.3
167	training	50	a	47.3	a	55.8	a	43.6
168	training	50	a	45.3	a	46.1	a	31.7
169	training	50	a	48.8	a	51.4	a	54.3
170	training	50	a	56.1	a	41.7	a	44.9
171	test	80	a	75.0	a	70.9	a	73.4
172	test	80	a	73.1	a	71.4	a	74.7
173	test	80	a	76.4	a	69.6	a	72.9
174	test	80	a	75.2	a	69.2	a	69.5
175	test	80	a	76.6	a	74.5	a	63.0
176	test	80	a	67.2	a	65.4	a	45.3
177	test	80	a	75.1	a	69.3	a	65.4
178	test	80	a	69.3	a	72.3	a	26.9
179	test	80	a	68.9	a	73.3	a	66.7
180	training	80	a	70.9	a	68.0	a	26.3
181	training	80	a	68.5	a	69.3	a	33.0
182	training	80	a	76.8	a	65.9	a	60.8
183	training	80	a	76.0	a	66.5	a	68.8
184	training	80	a	74.4	a	66.9	a	13.3
185	training	80	a	77.0	a	68.9	a	57.3
186	training	80	a	74.3	a	72.6	a	71.7
187	training	80	a	71.4	a	73.7	a	65.3
188	training	80	a	70.4	a	71.6	a	66.8
189	training	80	a	69.9	a	70.7	a	73.3
190	training	80	a	66.5	a	72.5	a	68.8
191	training	80	a	74.7	a	59.9	a	70.8
192	training	80	a	72.5	a	66.7	a	71.2
193	training	80	a	74.4	a	72.4	a	68.0
194	training	80	a	69.4	a	67.7	a	63.7

195	training	80	a	61.3	a	74.6	a	74.8
196	training	80	a	59.5	a	76.3	a	75.4
197	training	80	a	66.7	a	77.6	a	76.4
198	training	80	a	64.3	a	75.9	a	68.7
199	training	80	a	61.8	a	68.0	a	61.8
200	training	80	a	63.3	a	75.9	a	72.4
201	training	80	a	68.1	a	74.0	a	74.8
202	training	80	a	67.5	a	76.8	a	75.4
203	training	80	a	75.6	a	77.2	a	76.4
204	training	80	a	76.1	a	73.8	a	68.7
205	training	100	a	73.8	a	62.7	a	61.8

Table S3. Classification and regression results of the analysis of the RP full scan data of the honey samples adulterated with rice, beet and high fructose corn syrup. For the classification, the adulterated class contained all of the samples with 5, 10, 20, 50 and 80% addition of the respective syrup (p = pure, a = adulterated, HFCS = high fructose corn syrup).

Honey no.	Dataset	True syrup proportion [%]	Classification rice syrup	Proportion estimation rice syrup [%]	Classification beet syrup	Proportion estimation beet syrup [%]	Classification HFCS	Proportion estimation HFCS [%]
1	test	0	p	3.15	p	3.27	a	24.3
2	test	0	p	2.57	p	2.55	p	16.5
3	test	0	p	3.09	p	1.18	p	10.7
4	test	0	p	1.65	p	0.59	p	8.11
5	test	0	p	4.86	p	0.63	p	7.53
6	test	0	p	4.70	p	2.15	p	11.9
7	test	0	p	4.88	p	5.29	a	18.6
8	test	0	p	3.29	p	0.71	p	5.42
9	test	0	p	2.21	p	0.79	p	9.28
10	training	0	p	2.32	p	0.58	a	10.6
11	training	0	p	1.87	p	1.46	a	6.87
12	training	0	p	5.20	p	0.85	a	8.23
13	training	0	p	2.87	p	0.85	p	6.62
14	training	0	p	2.31	p	0.80	p	9.89
15	training	0	p	3.29	p	0.71	p	6.45
16	training	0	p	3.16	p	1.28	p	8.55
17	training	0	p	3.96	p	1.97	a	14.6
18	training	0	p	5.66	p	2.47	a	9.52
19	training	0	a	6.26	p	2.17	a	13.4
20	training	0	p	1.84	p	1.05	p	6.84
21	training	0	p	3.16	p	0.75	a	15.5
22	training	0	p	2.28	p	0.91	a	6.21
23	training	0	p	3.74	p	0.82	p	5.41
24	training	0	p	4.18	p	0.82	p	4.67
25	training	0	p	2.75	p	1.82	p	5.94
26	training	0	p	3.23	p	1.20	p	7.35
27	training	0	p	3.43	p	1.19	a	6.55
28	training	0	p	3.78	p	1.42	a	10.2
29	training	0	p	4.05	p	5.88	a	14.2
30	training	0	p	6.56	p	0.96	a	7.18
31	training	0	p	1.97	p	0.90	a	10.8
32	training	0	p	2.56	a	20.5	a	8.51
33	training	0	p	3.74	p	1.51	a	6.60
34	training	0	p	2.29	p	1.84	p	5.82
35	test	5	a	8.79	a	8.27	a	18.6
36	test	5	a	7.55	a	6.77	a	18.8
37	test	5	a	9.12	a	6.81	a	16.1
38	test	5	a	6.69	a	5.92	a	8.12
39	test	5	a	9.41	a	7.35	a	17.4
40	test	5	a	8.91	a	6.68	a	15.3
41	test	5	a	7.86	a	8.85	a	17.6
42	test	5	a	8.31	a	6.63	a	13.9

43	test	5	a	7.54	a	6.53	a	12.1
44	training	5	a	7.10	a	6.12	a	10.8
45	training	5	a	6.74	a	5.98	a	14.0
46	training	5	a	7.50	a	6.59	a	9.91
47	training	5	a	7.14	a	7.00	a	15.6
48	training	5	a	7.14	a	6.30	a	13.3
49	training	5	a	6.57	a	6.49	a	16.4
50	training	5	a	9.91	a	6.55	a	15.6
51	training	5	a	9.59	a	7.81	a	11.0
52	training	5	a	8.68	a	7.30	a	9.74
53	training	5	a	10.9	a	6.24	a	8.14
54	training	5	a	12.0	a	7.73	a	8.28
55	training	5	a	6.52	a	10.0	a	12.8
56	training	5	a	9.48	a	6.67	a	20.0
57	training	5	a	10.0	a	7.20	a	7.83
58	training	5	a	10.6	a	7.42	a	15.5
59	training	5	a	9.68	a	6.59	a	13.9
60	training	5	a	10.6	a	11.2	a	43.5
61	training	5	a	8.91	a	7.22	a	9.58
62	training	5	a	6.24	a	5.98	a	8.11
63	training	5	a	7.43	a	8.38	a	9.89
64	training	5	a	7.56	a	6.23	a	8.48
65	training	5	a	7.45	a	5.63	a	8.24
66	training	5	a	7.03	a	6.23	a	8.70
67	training	5	a	8.36	a	6.94	a	11.1
68	training	5	a	8.54	a	6.10	a	9.31
69	test	10	a	10.7	a	13.9	a	20.0
70	test	10	a	8.82	a	10.4	a	27.1
71	test	10	a	11.8	a	11.9	a	14.8
72	test	10	a	9.63	a	11.1	a	13.7
73	test	10	a	29.3	a	12.7	a	15.1
74	test	10	a	14.9	p	1.16	a	20.1
75	test	10	a	10.3	a	13.6	a	23.6
76	test	10	a	14.9	a	9.83	a	17.6
77	test	10	a	13.5	a	10.5	a	69.5
78	training	10	a	9.94	a	9.52	a	21.7
79	training	10	a	9.11	a	10.7	a	20.4
80	training	10	a	15.5	a	11.8	a	20.0
81	training	10	a	24.2	a	9.98	a	19.5
82	training	10	a	22.9	a	10.4	a	12.6
83	training	10	a	12.8	a	11.3	a	13.4
84	training	10	a	9.64	a	14.3	a	8.94
85	training	10	a	15.7	a	8.74	a	17.3
86	training	10	a	6.22	a	11.2	a	15.2
87	training	10	a	16.6	a	10.6	a	17.9
88	training	10	a	10.8	a	12.1	a	13.2
89	training	10	a	10.1	a	13.4	a	12.6
90	training	10	a	11.8	a	11.5	a	9.38
91	training	10	a	9.16	a	13.6	a	16.6
92	training	10	a	12.1	a	13.0	a	10.6
93	training	10	a	9.78	a	11.5	a	10.2

94	training	10	a	10.4	a	11.4	a	19.7
95	training	10	a	11.3	a	10.7	a	14.5
96	training	10	a	9.74	a	9.23	a	13.3
97	training	10	a	9.12	a	11.9	a	10.1
98	training	10	a	11.2	a	10.1	a	12.0
99	training	10	a	12.9	a	11.0	a	12.1
100	training	10	a	8.64	a	11.8	a	14.2
101	training	10	a	10.4	a	10.4	a	10.3
102	training	10	a	12.8	a	12.4	a	12.2
103	test	20	a	15.5	a	19.7	a	27.7
104	test	20	a	14.1	a	19.9	a	60.3
105	test	20	a	20.2	a	22.8	a	31.9
106	test	20	a	18.2	a	27.7	a	25.3
107	test	20	a	27.9	a	23.0	a	34.2
108	test	20	a	16.5	a	16.8	a	18.2
109	test	20	a	18.3	a	23.2	a	29.3
110	test	20	a	24.0	a	23.3	a	20.3
111	test	20	a	15.8	a	26.1	a	21.8
112	training	20	a	19.2	a	17.0	a	22.8
113	training	20	a	18.7	a	16.8	a	47.0
114	training	20	a	28.8	a	18.3	a	19.9
115	training	20	a	41.4	a	22.5	a	17.5
116	training	20	a	24.4	a	24.2	a	16.3
117	training	20	a	21.0	a	23.3	a	27.3
118	training	20	a	18.9	a	19.6	a	22.5
119	training	20	a	28.1	a	21.1	a	26.6
120	training	20	a	12.9	a	20.5	a	17.7
121	training	20	a	15.4	a	24.3	a	27.4
122	training	20	a	42.1	a	25.9	a	14.2
123	training	20	a	16.7	a	25.4	a	20.8
124	training	20	a	23.8	a	25.3	a	20.9
125	training	20	a	16.9	a	24.8	a	32.6
126	training	20	a	19.4	a	19.5	a	24.4
127	training	20	a	12.8	a	15.6	a	29.4
128	training	20	a	17.5	a	17.3	a	15.9
129	training	20	a	21.3	a	19.2	a	21.8
130	training	20	a	17.4	a	18.4	a	14.4
131	training	20	a	19.0	a	22.2	a	16.9
132	training	20	a	12.7	a	26.6	a	17.1
133	training	20	a	15.7	a	20.9	a	19.7
134	training	20	a	17.8	a	23.6	a	15.2
135	training	20	a	20.6	a	20.4	a	21.4
136	training	20	a	30.1	a	7.8	a	19.3
137	test	50	a	42.5	a	46.5	a	45.6
138	test	50	a	40.8	a	50.4	a	46.2
139	test	50	a	51.9	a	48.9	a	30.2
140	test	50	a	39.3	a	49.5	a	48.6
141	test	50	a	55.9	a	63.9	a	49.3
142	test	50	a	42.6	a	50.1	a	33.4
143	test	50	a	35.3	a	43.3	a	44.7
144	test	50	a	58.2	a	51.0	a	59.7

145	test	50	a	44.3	a	51.7	a	47.9
146	training	50	a	43.5	a	59.2	a	63.1
147	training	50	a	59.8	a	43.9	a	52.9
148	training	50	a	49.5	a	45.9	a	52.1
149	training	50	a	51.9	a	49.7	a	63.7
150	training	50	a	54.2	a	53.8	a	46.1
151	training	50	a	56.5	a	49.6	a	47.8
152	training	50	a	42.7	a	53.2	a	41.8
153	training	50	a	40.8	a	51.2	a	45.8
154	training	50	a	52.9	a	56.9	a	39.6
155	training	50	a	34.0	a	59.8	a	43.1
156	training	50	a	50.9	a	51.5	a	40.8
157	training	50	a	47.7	a	57.3	a	53.1
158	training	50	a	57.7	a	42.0	a	49.0
159	training	50	a	38.1	a	56.8	a	58.8
160	training	50	a	55.7	a	57.7	a	43.5
161	training	50	a	55.0	a	43.7	a	47.0
162	training	50	a	46.2	a	47.8	a	49.8
163	training	50	a	40.5	a	49.1	a	20.4
164	training	50	a	33.5	a	58.2	a	40.2
165	training	50	a	39.6	a	53.3	a	32.6
166	training	50	a	39.0	a	48.9	a	52.3
167	training	50	a	49.3	a	54.0	a	50.8
168	training	50	a	39.5	a	55.0	a	54.2
169	training	50	a	46.2	a	59.4	a	62.2
170	training	50	a	61.5	a	57.3	a	38.6
171	test	80	a	72.0	a	66.9	a	66.8
172	test	80	a	63.8	a	75.5	a	67.3
173	test	80	a	77.8	a	72.5	a	74.4
174	test	80	a	75.7	a	67.4	a	61.7
175	test	80	a	79.5	a	76.4	a	77.1
176	test	80	a	75.1	a	69.8	a	69.4
177	test	80	a	69.4	a	71.1	a	58.6
178	test	80	a	73.5	a	73.1	a	34.8
179	test	80	a	71.8	a	76.9	a	71.1
180	training	80	a	76.7	a	70.9	a	22.4
181	training	80	a	73.7	a	68.2	a	31.6
182	training	80	a	79.7	a	72.6	a	69.5
183	training	80	a	78.6	a	68.8	a	67.9
184	training	80	a	79.1	a	70.6	a	12.8
185	training	80	a	78.6	a	75.2	a	60.8
186	training	80	a	74.7	a	70.0	a	75.3
187	training	80	a	75.8	a	73.0	a	73.0
188	training	80	a	67.2	a	70.4	a	52.0
189	training	80	a	75.2	a	76.0	a	75.6
190	training	80	a	75.8	a	74.1	a	68.2
191	training	80	a	76.3	a	67.2	a	73.8
192	training	80	a	75.0	a	73.1	a	73.1
193	training	80	a	75.7	a	77.7	a	67.0
194	training	80	a	77.5	a	72.8	a	73.2
195	training	80	a	73.6	a	77.3	a	64.9

196	training	80	a	66.4	a	74.7	a	61.6
197	training	80	a	72.0	a	78.7	a	69.3
198	training	80	a	64.2	a	78.5	a	72.2
199	training	80	a	66.5	a	77.3	a	73.5
200	training	80	a	67.2	a	75.7	a	75.2
201	training	80	a	73.4	a	77.6	a	78.7
202	training	80	a	69.0	a	76.3	a	69.4
203	training	80	a	72.9	a	75.8	a	67.5
204	training	80	a	78.9	a	74.3	a	77.9
205	training	100	a	78.6	a	71.1	a	74.0

Table S4. Classification and regression results of the analysis of the fused full scan and fragment data of the honey samples adulterated with rice, beet and high fructose corn syrup. For the classification, the adulterated class contained all of the samples with 5, 10, 20, 50 and 80% addition of the respective syrup (p = pure, a = adulterated, HFCS = high fructose corn syrup).

Honey no.	Dataset	True syrup proportion [%]	Classification rice syrup	Proportion estimation rice syrup [%]	Classification beet syrup	Proportion estimation beet syrup [%]	Classification HFCS	Proportion estimation HFCS [%]
1	test	0	p	1.53	p	2.41	p	21.1
2	test	0	p	1.36	p	1.48	p	13.0
3	test	0	p	1.35	p	0.95	p	9.98
4	test	0	p	0.92	p	0.97	p	6.56
5	test	0	p	2.68	p	1.12	p	6.24
6	test	0	p	2.20	p	2.63	p	10.6
7	test	0	p	3.02	p	3.03	p	13.1
8	test	0	p	1.42	p	0.44	p	3.83
9	test	0	p	0.36	p	0.57	p	6.82
10	training	0	p	0.99	p	0.22	a	8.91
11	training	0	p	0.68	p	1.28	p	6.57
12	training	0	p	1.86	p	0.77	p	6.88
13	training	0	p	1.42	p	1.60	p	5.93
14	training	0	p	0.81	p	0.31	p	6.43
15	training	0	p	0.87	p	0.51	p	5.03
16	training	0	p	0.66	p	1.21	p	5.98
17	training	0	p	1.48	p	1.58	a	12.4
18	training	0	p	2.42	p	1.39	a	8.08
19	training	0	p	2.54	p	1.37	a	11.9
20	training	0	p	0.74	p	0.91	p	5.19
21	training	0	p	0.96	p	0.66	a	14.3
22	training	0	p	0.50	p	0.62	p	5.03
23	training	0	p	1.73	p	0.58	p	5.16
24	training	0	p	2.92	p	0.38	p	3.70
25	training	0	p	0.68	p	1.30	p	3.98
26	training	0	p	1.98	p	0.80	p	5.23
27	training	0	p	1.51	p	0.85	p	5.66
28	training	0	p	1.54	p	0.78	p	11.2
29	training	0	p	1.25	p	3.26	a	11.4
30	training	0	p	2.30	p	1.45	a	7.14
31	training	0	p	1.12	p	0.79	a	9.30
32	training	0	p	0.66	a	19.4	a	6.66
33	training	0	p	1.61	p	1.81	a	6.67
34	training	0	p	0.85	p	1.18	P	8.13
35	test	5	a	9.45	a	7.83	a	13.9
36	test	5	a	8.32	a	6.87	a	15.8
37	test	5	a	8.77	a	6.66	a	18.2
38	test	5	a	7.10	a	6.40	a	9.22
39	test	5	a	8.68	a	7.07	a	13.5
40	test	5	a	9.05	a	6.71	a	13.7
41	test	5	a	9.17	a	8.20	a	13.1

42	test	5	a	7.68	a	6.78	a	11.8
43	test	5	a	7.67	a	6.52	a	10.1
44	training	5	a	8.04	a	6.50	a	9.69
45	training	5	a	7.65	a	6.19	a	11.9
46	training	5	a	8.44	a	6.58	a	9.26
47	training	5	a	7.93	a	7.56	a	13.0
48	training	5	a	7.34	a	7.20	a	14.3
49	training	5	a	8.65	a	6.35	a	15.1
50	training	5	a	9.15	a	6.08	a	12.9
51	training	5	a	10.3	a	6.96	a	9.59
52	training	5	a	8.11	a	6.84	a	9.15
53	training	5	a	11.3	a	5.89	a	6.78
54	training	5	a	10.9	a	7.99	a	7.01
55	training	5	a	7.95	a	10.1	a	11.9
56	training	5	a	9.46	a	6.42	a	15.1
57	training	5	a	10.0	a	6.67	a	7.73
58	training	5	a	10.1	a	7.21	a	13.6
59	training	5	a	10.6	a	6.84	a	11.7
60	training	5	a	10.0	a	8.98	a	33.2
61	training	5	a	9.05	a	6.59	a	8.86
62	training	5	a	7.76	a	6.66	a	7.36
63	training	5	a	8.00	a	9.19	a	10.5
64	training	5	a	8.41	a	7.56	a	8.16
65	training	5	a	8.22	a	6.46	a	8.07
66	training	5	a	8.00	a	7.22	a	8.16
67	training	5	a	8.54	a	8.57	a	12.1
68	training	5	a	9.29	a	6.51	a	10.65
69	test	10	a	12.5	a	13.2	a	16.2
70	test	10	a	10.6	a	10.4	a	22.7
71	test	10	a	12.0	a	10.8	a	15.0
72	test	10	a	10.2	a	11.2	a	12.6
73	test	10	a	25.5	a	13.1	a	15.1
74	test	10	a	13.4	p	1.13	a	18.0
75	test	10	a	12.0	a	11.4	a	19.3
76	test	10	a	14.9	a	10.6	a	16.2
77	test	10	a	13.6	a	10.1	a	69.8
78	training	10	a	9.60	a	9.02	a	23.7
79	training	10	a	9.86	a	10.7	a	21.3
80	training	10	a	15.6	a	13.3	a	19.7
81	training	10	a	26.4	a	10.7	a	21.4
82	training	10	a	23.8	a	11.5	a	14.1
83	training	10	a	13.2	a	12.1	a	13.1
84	training	10	a	10.7	a	13.9	a	8.82
85	training	10	a	15.1	a	8.32	a	16.6
86	training	10	a	7.25	a	10.3	a	16.6
87	training	10	a	15.2	a	9.86	a	15.8
88	training	10	a	11.4	a	12.8	a	12.0
89	training	10	a	10.5	a	13.9	a	10.5
90	training	10	a	12.0	a	11.1	a	9.09
91	training	10	a	11.8	a	13.3	a	14.8
92	training	10	a	12.1	a	10.8	a	10.0

93	training	10	a	11.5	a	10.0	a	9.12
94	training	10	a	11.6	a	10.7	a	18.1
95	training	10	a	11.7	a	9.57	a	13.0
96	training	10	a	9.67	a	9.62	a	12.7
97	training	10	a	10.4	a	10.9	a	10.4
98	training	10	a	11.8	a	10.0	a	12.4
99	training	10	a	12.1	a	11.9	a	13.5
100	training	10	a	9.48	a	13.3	a	13.9
101	training	10	a	10.3	a	10.5	a	9.44
102	training	10	a	12.2	a	11.4	a	8.77
103	test	20	a	18.4	a	17.5	a	25.3
104	test	20	a	15.0	a	19.0	a	60.0
105	test	20	a	19.2	a	23.6	a	29.9
106	test	20	a	18.3	a	24.4	a	27.5
107	test	20	a	27.9	a	23.3	a	31.0
108	test	20	a	18.0	a	19.1	a	18.0
109	test	20	a	18.2	a	22.1	a	27.0
110	test	20	a	23.4	a	21.2	a	21.4
111	test	20	a	16.3	a	25.1	a	21.0
112	training	20	a	18.3	a	17.3	a	52.5
113	training	20	a	16.9	a	18.7	a	18.1
114	training	20	a	33.6	a	19.7	a	16.9
115	training	20	a	38.0	a	25.6	a	18.4
116	training	20	a	21.2	a	27.6	a	30.6
117	training	20	a	19.5	a	20.0	a	25.1
118	training	20	a	18.6	a	17.9	a	28.6
119	training	20	a	24.9	a	20.2	a	19.6
120	training	20	a	15.5	a	20.5	a	25.1
121	training	20	a	17.5	a	24.2	a	14.4
122	training	20	a	39.7	a	24.4	a	21.5
123	training	20	a	19.4	a	23.8	a	22.5
124	training	20	a	24.4	a	23.5	a	34.6
125	training	20	a	18.3	a	22.3	a	26.1
126	training	20	a	19.5	a	18.4	a	25.2
127	training	20	a	15.2	a	16.2	a	16.2
128	training	20	a	19.0	-	-	a	25.2
129	training	20	a	23.9	a	18.3	a	15.2
130	training	20	a	18.3	a	19.4	a	16.8
131	training	20	a	16.6	a	22.1	a	20.5
132	training	20	a	13.2	a	26.5	a	19.3
133	training	20	a	17.5	a	20.7	a	16.2
134	training	20	a	18.9	a	25.1	a	22.7
135	training	20	a	21.9	a	19.4	a	18.6
136	training	20	a	30.2	a	8.9	a	16.5
137	test	50	a	46.2	a	47.7	a	49.7
138	test	50	a	43.8	a	47.8	a	49.1
139	test	50	a	52.2	a	48.6	a	23.1
140	test	50	a	40.0	a	49.5	a	51.2
141	test	50	a	55.8	a	62.5	a	52.0
142	test	50	a	43.6	a	50.0	a	31.0
143	test	50	a	40.0	a	45.1	a	47.2

144	test	50	a	51.6	a	50.8	a	60.0
145	test	50	a	45.4	a	48.9	a	50.7
146	training	50	a	43.9	a	54.8	a	65.0
147	training	50	a	59.4	a	43.1	a	54.8
148	training	50	a	52.0	a	46.9	a	54.1
149	training	50	a	51.9	a	48.1	a	65.3
150	training	50	a	54.4	a	55.7	a	48.3
151	training	50	a	56.6	a	48.6	a	50.3
152	training	50	a	43.8	a	51.6	a	44.2
153	training	50	a	44.8	a	55.1	a	44.7
154	training	50	a	52.5	a	61.1	a	44.8
155	training	50	a	36.2	a	60.7	a	46.5
156	training	50	a	47.6	a	51.6	a	42.1
157	training	50	a	46.9	a	53.3	a	54.3
158	training	50	a	51.1	a	40.6	a	52.0
159	training	50	a	39.0	a	50.8	a	60.7
160	training	50	a	55.5	a	52.8	a	45.2
161	training	50	a	53.8	a	47.4	a	45.9
162	training	50	a	47.3	a	50.1	a	44.8
163	training	50	a	42.0	a	49.4	a	21.4
164	training	50	a	34.4	a	62.8	a	40.3
165	training	50	a	40.7	a	55.9	a	35.1
166	training	50	a	37.3	a	51.8	a	49.4
167	training	50	a	48.8	a	53.6	a	52.5
168	training	50	a	39.8	a	47.4	a	54.9
169	training	50	a	48.0	a	52.4	a	39.7
170	training	50	a	60.2	a	50.1	a	47.9
171	test	80	a	74.0	a	69.9	a	69.4
172	test	80	a	67.3	a	75.0	a	69.8
173	test	80	a	77.6	a	74.1	a	75.1
174	test	80	a	76.0	a	69.4	a	63.7
175	test	80	a	79.3	a	76.9	a	75.3
176	test	80	a	74.5	a	70.1	a	64.3
177	test	80	a	71.9	a	71.8	a	61.7
178	test	80	a	73.8	a	73.9	a	28.1
179	test	80	a	71.4	a	77.3	a	70.7
180	training	80	a	76.4	a	70.0	a	29.5
181	training	80	a	73.8	a	67.5	a	35.2
182	training	80	a	79.3	a	69.3	a	67.6
183	training	80	a	78.2	a	69.9	a	68.8
184	training	80	a	78.8	a	68.6	a	14.4
185	training	80	a	79.0	a	75.0	a	60.7
186	training	80	a	74.7	a	72.4	a	75.6
187	training	80	a	75.3	a	74.7	a	71.9
188	training	80	a	68.8	a	74.1	a	57.6
189	training	80	a	75.6	a	75.1	a	76.2
190	training	80	a	74.7	a	75.3	a	69.6
191	training	80	a	76.4	a	65.8	a	74.5
192	training	80	a	75.1	a	71.5	a	73.5
193	training	80	a	76.3	a	75.5	a	68.5
194	training	80	a	76.6	a	71.9	a	72.7

195	training	80	a	71.6	a	77.7	a	64.1
196	training	80	a	67.4	a	76.1	a	62.1
197	training	80	a	73.0	a	78.8	a	69.0
198	training	80	a	66.2	a	78.8	a	73.3
199	training	80	a	67.5	a	75.3	a	75.1
200	training	80	a	67.0	a	77.2	a	77.2
201	training	80	a	72.8	a	77.6	a	78.8
202	training	80	a	68.3	a	77.7	a	69.1
203	training	80	a	75.5	a	76.9	a	68.1
204	training	80	a	78.8	a	74.7	a	77.5
205	training	100	a	78.0	a	70.9	a	68.3

Table S1. Classification and regression results of the analysis of the HILIC full scan and fragment data of the honey samples adulterated with rice, beet and high fructose corn syrup. For the classification, the adulterated class contained all of the samples with 5, 10, 20, 50 and 80% addition of the respective syrup (p = pure, a = adulterated).

Honey no.	Dataset	True syrup proportion [%]	Classification rice syrup	Proportion estimation rice syrup [%]	Classification beet syrup	Proportion estimation beet syrup [%]	Classification HFCS	Proportion estimation HFCS [%]
1	test	0	p	1.15	p	1.78	p	18.2
2	test	0	p	1.90	p	1.13	a	11.7
3	test	0	p	1.40	p	0.89	p	9.80
4	test	0	p	1.05	p	0.97	p	6.92
5	test	0	p	2.27	p	1.51	a	6.81
6	test	0	p	2.01	p	3.48	a	10.4
7	test	0	p	3.22	p	2.10	p	9.32
8	test	0	p	1.57	p	0.45	p	3.46
9	test	0	p	0.24	p	0.62	a	6.67
10	training	0	p	1.57	p	0.32	a	9.98
11	training	0	p	0.88	p	1.45	a	8.24
12	training	0	p	0.96	p	0.78	a	6.72
13	training	0	p	1.04	p	2.77	p	6.41
14	training	0	p	0.60	p	0.16	p	6.00
15	training	0	p	0.59	p	0.56	p	4.74
16	training	0	p	0.50	p	1.20	p	5.64
17	training	0	p	1.11	p	1.92	a	10.9
18	training	0	p	1.25	p	1.35	a	8.77
19	training	0	p	1.65	p	1.27	a	11.1
20	training	0	p	1.01	p	1.10	p	6.36
21	training	0	p	0.87	p	0.58	a	14.4
22	training	0	p	0.63	p	0.80	p	5.55
23	training	0	p	1.75	p	0.73	p	5.65
24	training	0	p	3.63	p	0.59	p	3.58
25	training	0	p	0.52	p	1.31	p	5.28
26	training	0	p	2.53	p	0.85	p	4.68
27	training	0	p	1.75	p	1.07	p	4.45
28	training	0	p	1.79	p	0.51	p	6.23
29	training	0	p	1.13	p	1.81	p	11.7
30	training	0	p	2.06	p	2.21	a	9.78
31	training	0	p	1.43	p	1.35	a	8.81
32	training	0	p	0.71	a	18.8	a	9.92
33	training	0	p	1.93	p	2.73	a	7.33
34	training	0	p	0.64	p	1.72	a	8.29
35	test	5	a	9.56	a	7.06	a	9.81
36	test	5	a	8.37	a	6.70	a	14.9
37	test	5	a	8.43	a	6.39	a	19.8
38	test	5	a	7.16	a	6.76	a	9.55
39	test	5	a	7.77	a	6.88	a	12.2
40	test	5	a	8.11	a	7.44	p	13.7
41	test	5	a	9.99	a	8.33	a	10.6
42	test	5	a	7.23	a	6.90	a	11.3

43	test	5	a	7.47	a	6.50	a	9.06
44	training	5	a	8.66	a	7.93	a	9.53
45	training	5	a	8.35	a	6.10	a	12.2
46	training	5	a	8.15	a	6.00	a	11.0
47	training	5	a	8.24	a	8.57	a	10.8
48	training	5	a	7.42	a	7.81	a	14.2
49	training	5	a	9.13	a	6.57	a	14.4
50	training	5	a	8.46	a	5.63	a	11.7
51	training	5	a	10.7	a	7.08	a	8.73
52	training	5	a	8.22	a	6.76	a	9.52
53	training	5	a	12.3	a	5.71	a	5.81
54	training	5	a	9.25	a	8.40	a	6.51
55	training	5	a	8.27	a	10.6	a	12.4
56	training	5	a	9.22	a	5.98	a	12.1
57	training	5	a	10.3	a	6.43	a	7.41
58	training	5	a	8.93	a	6.79	a	12.4
59	training	5	a	10.2	a	7.07	a	12.1
60	training	5	a	9.74	a	6.99	a	10.5
61	training	5	a	8.67	a	6.14	a	25.3
62	training	5	a	8.99	a	6.87	a	9.22
63	training	5	a	7.97	a	9.57	a	7.86
64	training	5	a	9.49	a	8.36	a	12.7
65	training	5	a	8.64	a	7.15	a	8.85
66	training	5	a	8.69	a	8.12	a	8.89
67	training	5	a	8.49	a	9.90	a	8.09
68	training	5	a	10.9	a	6.54	a	13.9
69	test	10	a	13.8	a	12.5	a	12.5
70	test	10	a	12.2	a	10.6	a	19.7
71	test	10	a	12.5	a	8.96	a	16.5
72	test	10	a	11.1	a	11.4	a	13.0
73	test	10	a	23.5	a	13.3	a	15.6
74	test	10	a	12.4	p	1.52	a	15.6
75	test	10	a	13.6	a	9.85	a	15.0
76	test	10	a	13.8	a	11.6	a	16.5
77	test	10	a	15.4	a	9.61	a	68.8
78	training	10	a	9.20	a	9.00	a	22.6
79	training	10	a	10.2	a	10.9	a	20.3
80	training	10	a	16.8	a	14.4	a	18.1
81	training	10	a	31.4	a	11.5	a	21.9
82	training	10	a	26.7	a	13.3	a	15.2
83	training	10	a	15.4	a	13.0	a	11.5
84	training	10	a	11.8	a	13.5	a	7.80
85	training	10	a	15.2	a	8.15	a	17.6
86	training	10	a	7.58	a	10.2	a	15.7
87	training	10	a	12.0	a	8.98	a	15.0
88	training	10	a	11.9	a	13.8	a	10.7
89	training	10	a	11.1	a	15.7	a	10.7
90	training	10	a	12.8	a	10.8	a	9.52
91	training	10	a	12.7	a	12.5	a	12.8
92	training	10	a	12.0	a	8.49	a	13.8
93	training	10	a	12.5	a	9.65	a	9.31

94	training	10	a	12.2	a	10.6	a	8.87
95	training	10	a	12.6	a	8.70	a	18.6
96	training	10	a	9.60	a	10.0	a	11.3
97	training	10	a	10.3	a	11.7	a	11.5
98	training	10	a	12.0	a	10.1	a	11.6
99	training	10	a	12.4	a	13.3	a	13.5
100	training	10	a	9.69	a	15.2	a	15.3
101	training	10	a	10.5	a	10.9	a	14.8
102	training	10	a	12.9	a	10.7	a	9.67
103	test	20	a	20.2	a	16.9	a	24.2
104	test	20	a	17.5	a	18.0	a	58.8
105	test	20	a	19.7	a	23.8	a	28.4
106	test	20	a	18.6	a	21.0	a	29.2
107	test	20	a	26.9	a	22.3	a	27.9
108	test	20	a	17.7	a	22.3	a	17.3
109	test	20	a	18.5	a	21.9	a	24.1
110	test	20	a	21.7	a	19.3	a	20.8
111	test	20	a	18.4	a	24.8	a	20.2
112	training	20	a	21.2	a	18.1	a	54.9
113	training	20	a	18.4	a	21.8	a	17.6
114	training	20	a	39.1	a	21.7	a	15.9
115	training	20	a	35.2	a	27.6	a	19.0
116	training	20	a	20.9	a	30.5	a	32.8
117	training	20	a	19.2	a	17.0	a	25.3
118	training	20	a	17.4	a	16.4	a	30.3
119	training	20	a	18.1	a	20.4	a	21.0
120	training	20	a	17.0	a	19.4	a	22.3
121	training	20	a	18.7	a	26.2	a	13.7
122	training	20	a	35.6	a	22.6	a	23.1
123	training	20	a	20.0	a	22.3	a	22.3
124	training	20	a	22.7	a	20.7	a	36.3
125	training	20	a	19.1	a	19.4	a	26.6
126	training	20	a	20.3	a	16.4	a	17.5
127	training	20	a	18.2	a	17.9	a	20.0
128	training	20	a	23.7	a	16.4	a	17.2
129	training	20	a	23.1	a	18.6	a	30.6
130	training	20	a	17.5	a	21.8	a	16.6
131	training	20	a	16.0	a	23.3	a	16.5
132	training	20	a	14.8	a	27.0	a	23.4
133	training	20	a	19.1	a	21.5	a	20.3
134	training	20	a	19.7	a	26.7	a	18.0
135	training	20	a	22.8	a	19.3	a	24.7
136	training	20	a	28.5	a	11.4	a	19.3
137	test	50	a	49.6	a	49.3	a	53.3
138	test	50	a	47.9	a	47.1	a	50.8
139	test	50	a	52.3	a	48.5	a	19.2
140	test	50	a	40.4	a	50.1	a	53.2
141	test	50	a	55.4	a	58.9	a	55.7
142	test	50	a	39.7	a	51.0	a	27.9
143	test	50	a	44.6	a	47.0	a	49.2
144	test	50	a	46.2	a	49.3	a	55.8

145	test	50	a	48.5	a	48.1	a	51.9
146	training	50	a	42.3	a	51.3	a	66.0
147	training	50	a	58.0	a	42.2	a	56.1
148	training	50	a	54.6	a	47.7	a	56.6
149	training	50	a	52.9	a	47.8	a	65.5
150	training	50	a	54.2	a	56.5	a	50.3
151	training	50	a	56.8	a	50.2	a	52.7
152	training	50	a	48.2	a	51.0	a	43.2
153	training	50	a	48.5	a	57.7	a	41.0
154	training	50	a	52.7	a	63.8	a	57.3
155	training	50	a	43.4	a	58.0	a	55.7
156	training	50	a	45.9	a	51.6	a	60.4
157	training	50	a	42.6	a	51.6	a	43.0
158	training	50	a	37.8	a	40.0	a	43.0
159	training	50	a	42.4	a	45.0	a	38.9
160	training	50	a	54.7	a	49.4	a	21.1
161	training	50	a	51.5	a	49.3	a	41.1
162	training	50	a	49.9	a	52.5	a	38.9
163	training	50	a	41.5	a	50.2	a	46.4
164	training	50	a	33.6	a	64.8	a	55.1
165	training	50	a	40.9	a	59.1	a	52.7
166	training	50	a	35.3	a	53.7	a	55.7
167	training	50	a	48.1	a	55.2	a	55.3
168	training	50	a	43.5	a	45.4	a	46.2
169	training	50	a	50.2	a	48.2	a	46.9
170	training	50	a	56.6	a	45.2	a	52.1
171	test	80	a	76.4	a	71.4	a	75.0
172	test	80	a	75.5	a	74.2	a	75.3
173	test	80	a	77.3	a	74.8	a	74.1
174	test	80	a	77.1	a	68.3	a	70.9
175	test	80	a	77.9	a	76.1	a	68.9
176	test	80	a	70.2	a	69.1	a	56.8
177	test	80	a	75.7	a	72.2	a	67.3
178	test	80	a	72.5	a	73.7	a	23.3
179	test	80	a	70.0	a	76.7	a	68.4
180	training	80	a	72.9	a	69.3	a	33.6
181	training	80	a	70.8	a	67.4	a	38.1
182	training	80	a	78.1	a	64.4	a	61.2
183	training	80	a	76.8	a	70.4	a	70.1
184	training	80	a	77.0	a	67.7	a	15.9
185	training	80	a	78.2	a	73.9	a	59.3
186	training	80	a	75.5	a	74.5	a	72.4
187	training	80	a	72.8	a	74.9	a	66.9
188	training	80	a	71.5	a	74.4	a	67.1
189	training	80	a	73.6	a	73.8	a	75.2
190	training	80	a	70.9	a	75.9	a	70.6
191	training	80	a	76.5	a	62.2	a	73.1
192	training	80	a	76.0	a	69.8	a	73.0
193	training	80	a	75.3	a	73.0	a	69.9
194	training	80	a	73.1	a	71.4	a	74.7
195	training	80	a	64.3	a	77.4	a	68.9

196	training	80	a	66.8	a	78.1	a	61.7
197	training	80	a	70.7	a	78.7	a	69.3
198	training	80	a	68.6	a	78.3	a	64.7
199	training	80	a	67.7	a	73.4	a	72.2
200	training	80	a	63.2	a	77.9	a	75.8
201	training	80	a	70.7	a	77.6	a	78.0
202	training	80	a	68.6	a	78.7	a	78.4
203	training	80	a	77.6	a	77.3	a	71.3
204	training	80	a	77.8	a	74.4	a	64.6
205	training	100	a	75.9	a	69.4	a	75.1

Table S2. Classification and regression results of the analysis of the RP full scan and fragment data of the honey samples adulterated with rice, beet and high fructose corn syrup. For the classification, the adulterated class contained all of the samples with 5, 10, 20, 50 and 80% addition of the respective syrup (a = adulterated, p = pure, HFCS = high fructose corn syrup).

Honey no.	Dataset	True syrup proportion [%]	Classification rice syrup	Proportion estimation rice syrup [%]	Classification beet syrup	Proportion estimation beet syrup [%]	Classification HFCS	Proportion estimation HFCS [%]
1	test	0	p	2.94	p	3.81	a	24.3
2	test	0	p	2.04	p	2.64	p	18.0
3	test	0	p	2.72	p	1.24	p	11.9
4	test	0	p	1.51	p	0.58	p	7.24
5	test	0	p	4.29	p	0.56	p	7.37
6	test	0	p	4.40	p	2.33	p	12.4
7	test	0	p	4.27	p	5.61	p	19.1
8	test	0	p	3.34	p	0.64	p	5.18
9	test	0	p	1.91	p	1.01	p	9.07
10	training	0	p	2.39	p	0.62	a	9.88
11	training	0	p	1.77	p	1.73	a	7.14
12	training	0	p	4.06	p	0.88	a	8.61
13	training	0	p	2.23	p	1.07	p	6.89
14	training	0	p	2.01	p	0.86	p	9.26
15	training	0	p	3.03	p	0.84	p	6.98
16	training	0	p	3.04	p	1.31	p	8.38
17	training	0	p	3.66	p	2.04	a	13.1
18	training	0	p	4.92	p	2.16	a	9.00
19	training	0	a	5.69	p	2.62	a	13.3
20	training	0	p	2.28	p	1.01	p	6.45
21	training	0	p	2.74	p	1.00	a	15.4
22	training	0	p	2.05	p	0.87	p	5.70
23	training	0	p	4.41	p	0.91	p	4.92
24	training	0	p	5.01	p	0.87	p	4.66
25	training	0	p	2.49	p	1.97	p	5.01
26	training	0	p	3.01	p	1.04	p	7.17
27	training	0	p	3.39	p	1.22	a	5.87
28	training	0	p	3.50	p	1.30	a	10.7
29	training	0	p	3.32	p	5.09	a	13.4
30	training	0	p	5.67	p	1.04	a	7.11
31	training	0	p	1.81	p	1.25	a	10.9
32	training	0	p	2.03	a	20.0	a	7.85
33	training	0	p	3.55	p	1.47	a	6.81
34	training	0	p	2.28	p	1.83	a	8.77
35	test	5	a	8.39	a	8.51	a	19.2
36	test	5	a	7.35	a	6.85	a	19.1
37	test	5	a	9.00	a	6.91	a	16.7
38	test	5	a	6.74	a	6.23	a	8.11
39	test	5	a	9.13	a	7.23	a	16.4
40	test	5	a	8.67	a	6.80	a	14.0
41	test	5	a	7.72	a	8.35	a	17.5

42	test	5	a	7.61	a	6.59	a	13.9
43	test	5	a	7.34	a	6.58	a	12.4
44	training	5	a	6.63	a	6.63	a	10.6
45	training	5	a	6.75	a	6.18	a	12.7
46	training	5	a	7.72	a	6.68	a	9.36
47	training	5	a	7.19	a	6.94	a	15.8
48	training	5	a	6.96	a	6.53	a	14.0
49	training	5	a	7.07	a	6.21	a	16.4
50	training	5	a	9.54	a	6.54	a	14.9
51	training	5	a	9.24	a	7.74	a	10.3
52	training	5	a	8.20	a	7.08	a	9.83
53	training	5	a	10.1	a	6.29	a	7.70
54	training	5	a	12.0	a	7.57	a	8.36
55	training	5	a	6.71	a	9.80	a	13.9
56	training	5	a	9.63	a	6.59	a	20.0
57	training	5	a	10.7	a	6.93	a	7.87
58	training	5	a	11.0	a	7.55	a	15.1
59	training	5	a	10.1	a	6.41	a	13.3
60	training	5	a	11.1	a	10.8	a	43.3
61	training	5	a	9.25	a	6.88	a	9.17
62	training	5	a	6.32	a	6.28	a	8.32
63	training	5	a	7.78	a	8.26	a	10.1
64	training	5	a	6.95	a	6.64	a	8.81
65	training	5	a	7.51	a	6.09	a	8.09
66	training	5	a	6.65	a	6.45	a	8.60
67	training	5	a	8.24	a	7.25	a	10.6
68	training	5	a	8.23	a	6.20	a	7.47
69	test	10	a	10.7	a	13.7	a	20.1
70	test	10	a	8.74	a	10.2	a	28.7
71	test	10	a	11.8	a	12.0	a	15.1
72	test	10	a	9.39	a	11.1	a	13.6
73	test	10	a	27.4	a	12.8	a	15.3
74	test	10	a	14.8	p	1.53	a	20.8
75	test	10	a	10.3	a	12.7	a	24.4
76	test	10	a	14.8	a	9.91	a	18.1
77	test	10	a	12.5	a	10.4	a	69.4
78	training	10	a	9.47	a	9.06	a	21.7
79	training	10	a	9.01	a	10.5	a	20.0
80	training	10	a	15.4	a	11.9	a	19.5
81	training	10	a	25.4	a	10.6	a	19.7
82	training	10	a	22.0	a	10.3	a	14.9
83	training	10	a	12.0	a	11.2	a	14.8
84	training	10	a	10.2	a	14.4	a	9.38
85	training	10	a	15.1	a	8.63	a	16.5
86	training	10	a	6.09	a	10.7	a	14.6
87	training	10	a	17.0	a	10.4	a	17.6
88	training	10	a	10.7	a	12.1	a	13.3
89	training	10	a	10.4	a	13.0	a	12.2
90	training	10	a	11.8	a	11.4	a	9.77
91	training	10	a	9.54	a	13.8	a	17.8
92	training	10	a	11.9	a	12.1	a	10.5

93	training	10	a	9.29	a	10.9	a	10.6
94	training	10	a	11.0	a	10.5	a	18.6
95	training	10	a	11.6	a	9.68	a	14.9
96	training	10	a	9.39	a	9.13	a	13.0
97	training	10	a	9.34	a	11.7	a	10.8
98	training	10	a	12.1	a	10.1	a	12.1
99	training	10	a	11.9	a	11.1	a	12.3
100	training	10	a	8.52	a	12.2	a	13.3
101	training	10	a	9.04	a	10.0	a	10.5
102	training	10	a	12.9	a	12.2	a	9.42
103	test	20	a	15.7	a	18.5	a	27.4
104	test	20	a	13.3	a	19.8	a	60.9
105	test	20	a	19.0	a	23.3	a	30.8
106	test	20	a	17.9	a	27.5	a	24.2
107	test	20	a	27.6	a	23.6	a	33.8
108	test	20	a	17.4	a	16.9	a	19.0
109	test	20	a	18.0	a	22.4	a	29.5
110	test	20	a	23.1	a	23.0	a	21.5
111	test	20	a	15.3	a	24.9	a	21.3
112	training	20	a	17.6	a	16.6	a	46.5
113	training	20	a	16.8	a	16.7	a	18.9
114	training	20	a	30.5	a	18.3	a	17.4
115	training	20	a	40.3	a	22.6	a	17.2
116	training	20	a	23.0	a	25.2	a	28.0
117	training	20	a	19.0	a	22.9	a	24.2
118	training	20	a	19.3	a	19.3	a	25.2
119	training	20	a	28.3	a	20.7	a	17.4
120	training	20	a	13.3	a	20.5	a	25.7
121	training	20	a	16.0	a	23.9	a	14.2
122	training	20	a	41.7	a	26.8	a	21.0
123	training	20	a	17.0	a	25.2	a	20.7
124	training	20	a	24.0	a	24.6	a	32.4
125	training	20	a	16.7	a	24.6	a	25.6
126	training	20	a	18.9	a	19.7	a	25.6
127	training	20	a	12.9	a	15.0	a	16.1
128	training	20	a	18.0	-	-	a	21.1
129	training	20	a	23.5	a	18.1	a	13.9
130	training	20	a	17.8	a	17.9	a	17.5
131	training	20	a	16.8	a	21.9	a	16.5
132	training	20	a	12.2	a	25.7	a	19.1
133	training	20	a	16.0	a	20.7	a	15.0
134	training	20	a	17.5	a	22.9	a	21.2
135	training	20	a	20.2	a	19.9	a	19.3
136	training	20	a	30.7	a	7.5	a	17.4
137	test	50	a	44.5	a	46.0	a	44.7
138	test	50	a	41.0	a	49.4	a	45.6
139	test	50	a	52.0	a	48.5	a	28.2
140	test	50	a	39.1	a	49.8	a	48.2
141	test	50	a	56.2	a	64.6	a	49.5
142	test	50	a	45.4	a	50.6	a	32.7
143	test	50	a	37.4	a	43.1	a	43.9

144	test	50	a	55.1	a	51.0	a	60.2
145	test	50	a	44.4	a	49.6	a	47.5
146	training	50	a	45.3	a	58.7	a	63.2
147	training	50	a	61.6	a	43.1	a	51.8
148	training	50	a	51.5	a	46.1	a	53.3
149	training	50	a	52.1	a	49.4	a	64.4
150	training	50	a	54.7	a	53.5	a	46.5
151	training	50	a	56.3	a	49.1	a	47.9
152	training	50	a	44.1	a	53.0	a	40.4
153	training	50	a	42.4	a	51.5	a	46.1
154	training	50	a	54.3	a	57.7	a	43.4
155	training	50	a	33.1	a	60.0	a	45.0
156	training	50	a	48.0	a	52.1	a	41.3
157	training	50	a	48.7	a	56.2	a	52.1
158	training	50	a	57.4	a	43.1	a	47.5
159	training	50	a	37.9	a	56.9	a	61.1
160	training	50	a	56.9	a	57.0	a	44.7
161	training	50	a	55.9	a	44.6	a	46.2
162	training	50	a	47.0	a	48.0	a	48.6
163	training	50	a	42.7	a	48.5	a	19.4
164	training	50	a	32.8	a	59.6	a	39.7
165	training	50	a	39.4	a	55.4	a	31.5
166	training	50	a	38.6	a	51.2	a	53.4
167	training	50	a	47.8	a	53.4	a	51.9
168	training	50	a	37.7	a	51.4	a	55.3
169	training	50	a	47.2	a	59.4	a	60.9
170	training	50	a	62.3	a	57.3	a	41.2
171	test	80	a	72.9	a	67.5	a	66.1
172	test	80	a	63.7	a	75.7	a	67.1
173	test	80	a	77.3	a	72.2	a	75.1
174	test	80	a	75.1	a	67.8	a	56.7
175	test	80	a	79.5	a	77.1	a	78.0
176	test	80	a	75.6	a	70.2	a	67.7
177	test	80	a	70.4	a	70.6	a	57.7
178	test	80	a	73.9	a	74.2	a	32.8
179	test	80	a	71.3	a	77.4	a	70.8
180	training	80	a	76.7	a	70.9	a	23.4
181	training	80	a	74.6	a	67.3	a	29.9
182	training	80	a	79.6	a	72.8	a	71.0
183	training	80	a	78.1	a	69.3	a	68.6
184	training	80	a	79.1	a	69.9	a	13.1
185	training	80	a	78.6	a	75.5	a	60.7
186	training	80	a	74.0	a	70.8	a	76.0
187	training	80	a	76.4	a	74.1	a	72.7
188	training	80	a	67.0	a	71.7	a	52.6
189	training	80	a	75.7	a	76.3	a	75.8
190	training	80	a	75.4	a	74.3	a	69.2
191	training	80	a	76.1	a	68.0	a	73.8
192	training	80	a	74.0	a	73.6	a	73.4
193	training	80	a	75.8	a	76.8	a	68.3
194	training	80	a	76.8	a	71.5	a	73.7

195	training	80	a	74.4	a	77.8	a	65.3
196	training	80	a	67.0	a	74.3	a	56.1
197	training	80	a	73.4	a	78.9	a	69.0
198	training	80	a	63.8	a	78.9	a	72.9
199	training	80	a	67.0	a	77.3	a	74.7
200	training	80	a	66.7	a	76.2	a	76.9
201	training	80	a	72.3	a	77.6	a	79.2
202	training	80	a	67.2	a	76.6	a	67.7
203	training	80	a	72.9	a	76.4	a	67.8
204	training	80	a	79.0	a	74.0	a	78.4
205	training	100	a	77.8	a	71.8	a	75.3

Table S3. RMSE for the prediction of the proportion of rice, beet and high fructose corn syrup in honey samples by the random forest regression models.

Data type	Syrup proportion [%]	RMSE rice syrup data [%]		RMSE beet syrup data [%]		RMSE high fructose corn syrup data [%]	
		Training	Test	Training	Test	Training	Test
HILIC full scan data	0	13	2.9	8.1	6.6	9.0	10
	5	9.2	4.0	4.6	2.7	25	8.4
	10	3.1	7.1	3.2	3.1	7.8	20
	20	5.7	3.4	14	3.8	14	14
	50	7.0	7.3	5.0	4.3	25	14
	80	5.7	8.8	5.5	9.9	11	24
HILIC full scan and fragment data	0	13	1.9	8.0	6.4	7.9	7.9
	5	7.9	3.1	5.0	1.9	22	7.8
	10	2.3	5.3	3.2	3.5	8.7	20
	20	4.0	2.8	11	2.6	14	14
	50	6.6	5.5	5.7	3.5	24	14
	80	4.8	6.8	4.9	7.6	10	22
RP full scan data	0	12	3.5	4.6	6.9	7.3	9.8
	5	7.2	3.5	4.8	1.9	27	9.1
	10	2.3	7.0	4.3	3.4	9.2	21
	20	6.8	4.3	10	3.9	12	15
	50	5.0	8.0	3.9	5.2	25	11
	80	4.6	8.0	5.0	7.5	9.3	21
RP full scan and fragment data	0	11	3.1	5.9	7.6	7.7	10
	5	6.5	3.0	3.2	1.6	27	8.9
	10	2.3	6.3	4.3	1.7	9.0	21
	20	6.3	4.3	9.0	4.0	11	15
	50	4.8	7.1	5.8	5.4	25	11
	80	5.0	7.8	4.5	7.6	9.1	22
Data fusion full scan data	0	12	1.7	6.4	7.2	7.1	7.9
	5	6.9	3.4	2.0	1.8	24	7.7
	10	2.1	5.8	3.8	1.7	8.6	21
	20	5.4	3.7	8.7	3.6	13	15
	50	5.2	6.0	6.0	4.6	25	12
	80	5.0	7.0	5.7	6.9	9.0	22
Data fusion full scan and fragment data	0	12	2.3	5.4	6.8	7.1	9.2
	5	7.1	3.7	4.4	2.0	26	8.4
	10	2.2	6.8	4.1	3.3	8.3	21
	20	6.3	3.9	11	3.6	12	15
	50	5.2	7.4	4.3	4.9	25	11
	80	4.7	7.7	5.1	7.8	9.6	21
